# Microbiota-dependent influence of prebiotics on the resilience of infant gut microbiota to amoxicillin/clavulanate perturbation in an *in vitro* colon model

**DOI:** 10.3389/fmicb.2023.1131953

**Published:** 2023-05-18

**Authors:** Martha F. Endika, David J. M. Barnett, Cynthia E. Klostermann, Henk A. Schols, Ilja C. W. Arts, John Penders, Arjen Nauta, Hauke Smidt, Koen Venema

**Affiliations:** ^1^Laboratory of Microbiology, Wageningen University & Research, Wageningen, Netherlands; ^2^Maastricht Centre for Systems Biology (MaCSBio), Maastricht University, Maastricht, Netherlands; ^3^Department of Medical Microbiology, Maastricht University Medical Center, Maastricht, Netherlands; ^4^Biobased Chemistry and Technology, Wageningen University and Research, Wageningen, Netherlands; ^5^Laboratory of Food Chemistry, Wageningen University and Research, Wageningen, Netherlands; ^6^FrieslandCampina, Amersfoort, Netherlands; ^7^Centre for Healthy Eating and Food Innovation (HEFI), Maastricht University—Campus Venlo, Venlo, Netherlands

**Keywords:** antibiotic, 2’-FL, GOS, pre-weaning, bacteria, recovery

## Abstract

Antibiotic exposure disturbs the developing infant gut microbiota. The capacity of the gut microbiota to recover from this disturbance (resilience) depends on the type of antibiotic. In this study, infant gut microbiota was exposed to a combination of amoxicillin and clavulanate (amoxicillin/clavulanate) in an *in vitro* colon model (TIM-2) with fecal-derived microbiota from 1-month-old (1-M; a mixed-taxa community type) as well as 3-month-old (3-M; *Bifidobacterium* dominated community type) breastfed infants. We investigated the effect of two common infant prebiotics, 2′-fucosyllactose (2’-FL) or galacto-oligosaccharides (GOS), on the resilience of infant gut microbiota to amoxicillin/clavulanate-induced changes in microbiota composition and activity. Amoxicillin/clavulanate treatment decreased alpha diversity and induced a temporary shift of microbiota to a community dominated by enterobacteria. Moreover, antibiotic treatment increased succinate and lactate in both 1- and 3-M colon models, while decreasing the production of short-chain (SCFA) and branched-chain fatty acids (BFCA). The prebiotic effect on the microbiota recovery depended on the fermenting capacity of antibiotic-exposed microbiota. In the 1-M colon model, the supplementation of 2’-FL supported the recovery of microbiota and restored the production of propionate and butyrate. In the 3-M colon model, GOS supplementation supported the recovery of microbiota and increased the production of acetate and butyrate.

## Introduction

The gut microbiota develops in the first years after birth, modulated strongly by the exposure to different dietary sources, antibiotics, and microbes from the environment (Tamburini et al., 2016). The exposure to antibiotics disturbs the development of microbiota throughout the first year of life, indicated by compositional deviations which are depending on the duration and type of antibiotic (Van Daele et al., 2022). A single course of 5–8 days of amoxicillin in infants (average age of 4 months) caused a long-term disruption for at least 6 months while recovering to a community characterized by an increase in the relative abundance of clostridia and a decrease in the relative abundance of bifidobacteria (Korpela et al., 2020). Most studies have only focused on amoxicillin (β-lactam antibiotic), whereas the use of a combination of amoxicillin and β-lactamase inhibitor clavulanate, another commonly prescribed antibiotic in infants, showed a different pattern of microbiota changes compared to amoxicillin (Korpela et al., 2020). Moreover, the exposure to amoxicillin or amoxicillin/clavulanate in the gut might increase the risk of being colonized by antimicrobial resistant bacteria (Korpela et al., 2020; Li et al., 2022).

Antibiotic exposure in early life is associated with an altered immune development and increased risk of infantile colic, wheezing, and overweight later in life (Saari et al., 2015; Korpela et al., 2017; Oosterloo et al., 2018, 2020); the antibiotic-induced perturbations in the development of gut microbiota are hypothesized to mediate this relation. As defined by Carvalho et al., antibiotic resilience is the capacity to recover from antibiotic exposure, while in practice this depends on which microbial system is considered, the disturbance applied (type, frequency, duration, and concentration of antibiotic), and the metric used to measure resilience (Carvalho et al., 1916). Strategies to recover the disturbed microbiota are needed to minimize antibiotic impact on both microbiota and immune development.

In the preweaning period, the gut of infants is exposed to nondigestible carbohydrates derived from the milk diet (human milk or fortified infant formula) that act as prebiotics. Prebiotics are defined as compounds that are selectively utilized by host microorganisms conferring a health benefit to the host, with galacto-oligosaccharides (GOS) and human milk oligosaccharides (HMOs) fitting this definition as infant prebiotics (Gibson et al., 2017). GOS are produced from lactose using β-galactosidase enzyme, resulting in a mixture varying in the degree of polymerization mainly between 2 and 8 with a terminal glucose and the remaining saccharide units being galactose, with differences in the linkages between monomers (Torres et al., 2010). On the other hand, HMOs are highly abundant and unique unconjugated complex carbohydrates in human milk, consisting of lactose at the reducing end, and composed of up to five different monosaccharide building blocks, including galactose, glucose, N-acetylglucosamine, fucose, and sialic acid (Bode, 2012). One of the most abundant HMOs, 2′-fucosyllactose (2’-FL), is present in high concentration in the milk of over 70% of Caucasian women who actively express *fucosyltransferase 2* (*FUT2*), also referred to as secretor mothers (Bode, 2015).

The supplementation of 2’-FL in infant formula has been shown to lower the abundance of opportunistic pathogens, stimulate the abundance of bifidobacteria, and support immune development to be more similar to that in breastfed infants (Goehring et al., 2016; Alliet et al., 2022). The supplementation of GOS in infant formula has been shown to stimulate bifidobacteria and lactobacilli, decrease fecal pH, increase the frequency of defecation, and soften the stools (Ben et al., 2008; Fanaro et al., 2009; Sierra et al., 2015). In adults receiving amoxicillin, the intake of GOS supported the recovery of bifidobacteria in *in vitro* and *in vivo* studies (Ladirat et al., 2014a,b), but this deserves further study in infants.

The use of controlled *in vitro* gut models offers a promising approach to investigate how a community responds to a disturbance in early life. A controlled gut fermentation system has been successfully used to simulate the growth of the infant gut microbiota and to investigate the effect of pharmaceuticals and diet-derived compounds during infancy (Le Blay et al., 2010; Wiese et al., 2018; Salli et al., 2019; Van den Abbeele et al., 2021; Natividad et al., 2022). A dynamic *in vitro* gut model (TIM-2) has been used to evaluate the effect of probiotics and antibiotics on healthy adult gut microbiota (Rehman et al., 2012), thus providing opportunities to conduct similar studies on infant gut microbiota.

The current study was conducted to study the resilience of the infant gut microbiota structure and activity in response to amoxicillin/clavulanate treatment, in the absence and presence of prebiotics, 2’-FL, or GOS.

## Materials and methods

### Antibiotic and prebiotics

Amoxicillin/clavulanate, a combination of amoxicillin trihydrate with potassium clavulanate (4:1; Sigma-Aldrich, Saint Louis, MO, United States), a broad-spectrum antibiotic, was used in this study. Prebiotics, 2′-fucosylactose (2’-FL) and galacto-oligosaccharides (GOS) were provided by FrieslandCampina Ingredients (Amersfoort, the Netherlands). Aequival 2’-FL powder is a high-purity human milk oligosaccharide product (94% 2’-FL on a dry matter basis). Purified Vivinal GOS was provided, containing 1.7% monomers, 6.6% dimers (allo-lactose, lactose and lactulose), and 91.7% GOS on a dry matter basis.

### Fecal sample collection and preparation

Infant fecal samples were collected at different ages during the Baby Carbs study. Briefly, the Baby Carbs study recruited mother-infant pairs living in the Netherlands. Inclusion criteria included vaginally delivered, full-term, exclusively breastfed infants who were not exposed to antibiotics (no antibiotic during labor and after birth). This study was exempted from medical research ethics committee approval for the collection of stool samples, after review by the Medical Ethical Reviewing Committee of Wageningen University. All parents provided written informed consent before the start of the sample collection. Participants collected fecal samples daily (consecutively during 1 week) from a diaper using a sterile spoon (Sampling Systems, Coleshill, United Kingdom) and kept the feces anaerobically in a sterile 50 mL collection tube inside a BD GasPak EZ anaerobe gas generating pouch (BD Diagnostics, Sparks, MD, United States). The fecal samples were stored in the home refrigerator (±4°C) for a maximum of 72 h prior to the collection by one of the researchers for transport to the lab. Previous study showed that refrigeration of feces at 4°C for short term storage (up to 72 h) did not alter fecal microbiota composition, compared to direct freezing to −80°C or cultures from fresh feces (Choo et al., 2015; Burz et al., 2019). These collections were scheduled up to two times per week. For transport to the lab, the refrigerated samples were put in an insulated bag containing frozen cooling elements, and upon arrival processed in the laboratory on the same day. Samples collected at the age of 1 month and 3 months were processed separately, as indicated below.

At the laboratory, the fecal samples from individual infants at the same age were pooled in a sterile 50 mL tube (CELLSTAR™ CELLreactor™ tube, Greiner Bio-One, Alphen aan den Rijn, the Netherlands) and weighed inside an anaerobic chamber (Bactron300-2, Sheldon Manufacturing, OR, United States; 96% N_2_, 4% H_2_). The fecal samples were diluted in a sterile anoxic solution (pH 6.5) containing 2.5 g/L KH_2_PO_4_, 4.5 g/L NaCl, 0.005 g/L FeSO_4_.7H_2_O, 0.05 g/L ox bile, 0.04 g/L Cysteine-HCl, and glycerol (final concentration of 10%, v/v). The fecal slurries (25% w/v) were mixed using vortex, then aliquoted into sterile 10 mL vials (La-Pha-Pack, Langerwehe, Germany). The vials were sealed with a sterile butyl rubber stopper with a crimp cap. The sealed fecal slurries were taken out from the anaerobic chamber and immediately snap-frozen in liquid nitrogen prior to storage at −80°C.

### Feeding media and dialysate solution composition

The simulated ileal efflux medium for infants (i-SIEM), a concentrated feeding medium modified from Cinquin et al. (2004), was used to simulate the compounds reaching the colon of infants in the preweaning period (milk diet). The i-SIEM consisted of the following components (per L): 24.0 g lactose (Merck, Darmstadt, Germany), 6 g tryptone (Oxoid, Basingstoke, United Kingdom), 6 g lactalbumin hydrolysate (Merck), 0.8 g ox bile (Merck), 15 g porcine gastric mucin (partially purified type III; Sigma-Aldrich), 0.6 g urea (Thermo Fisher Scientific, Waltham, MA, United States), 0.4 g cysteine HCl (Merck), 10 mL antifoam B emulsion (Sigma-Aldrich), salt solution (Tritium Mikrobiologie B.V., the Netherlands) containing 4.5 g, NaCl, 2.5 g K_2_HPO_4_.3H_2_O, 0.45 g CaCl_2_.2H_2_O, 0.005 FeSO_4_.7H_2_O, 0.01 g hemin, and 1 mL vitamin solution (Tritium Mikrobiologie B.V.) containing (per L) 1 mg menadione, 2 mg D-biotin, 0.5 mg vitamin B12, 10 mg D-pantothenate, 5 mg p-aminobenzoic acid, 4 mg thiamine HCl, and 5 mg nicotinamide adenine dinucleotide. All the components were autoclaved, except urea and vitamins, which were filter sterilized using sterile 0.2 μm membrane syringe filters (Advance Microdevices, Ambala Cantt, India). The porcine gastric mucin was centrifuged at 10,000 g for 10 min prior to sterilization. The prebiotic, either 2’-FL or GOS, was supplemented in the concentrated i-SIEM feeding medium at a concentration of 30 g/L, which was further diluted in the colon model. This corresponds to the prebiotic supplementation of 1.8 g/day.

In order to maintain the physiological concentration of electrolytes and metabolites, dialysis liquid was pumped at a rate of 1.5 mL/min through semi-permeable hollow-fiber membranes inside the lumen compartment of the TNO *in vitro* model of the colon (TIM-2; Minekus et al., 1999). Dialysis liquid (Tritium Mikrobiologie B.V.) contained (per L): 2.5 g K_2_HPO_4_.3H_2_O, 4.5 g NaCl, 0.5 g MgSO_4_.7H_2_O, 0.45 g CaCl_2_.2H_2_O, 0.05 g bile, 0.005 g FeSO_4_.7H_2_O, and 0.4 g cysteine HCl, plus 1 mL of a vitamin mixture as mentioned above. The pH of the dialysis liquid was adjusted to 6.0 with NaOH.

### Fecal inocula and TIM-2 fermentation procedures

In order to achieve sufficient volume of fecal material to inoculate each TIM-2 run with a consistent starting microbiota, the fecal slurries were pooled from four infants from each age, 1-month-old (1-M) or 3-month-old (3-M). The infant fecal slurries were selected for pooling based on their microbiota composition, as ascertained through 16S ribosomal RNA (rRNA) gene amplicon sequencing of fecal samples collected from 21 infants at 1 month of age and 23 infants at 3 months of age. Fecal microbiota was characterized based on the presence of dominant taxa (minimum relative abundance threshold of 50%) in the community (Supplementary Figure 1A).

In addition to the community characteristic, the selection of fecal slurries was also made based on the amount of fecal materials collected per individual infant (minimum of 4 g feces collected). The four infant fecal slurries selected for the pooled inocula in the 1-M colon model (1-M pool) were chosen for their highly similar and characteristically “mixed” and even microbial compositions, typical of this age. The fecal slurries selected for the pooled inocula in the 3-M colon model (3-M pool) were chosen because their compositions were highly similar and characteristically dominated by *Bifidobacterium* (relative abundance >50%). The microbiota composition of individual fecal slurries is provided in Supplementary Figure 1B.

**Figure 1:**
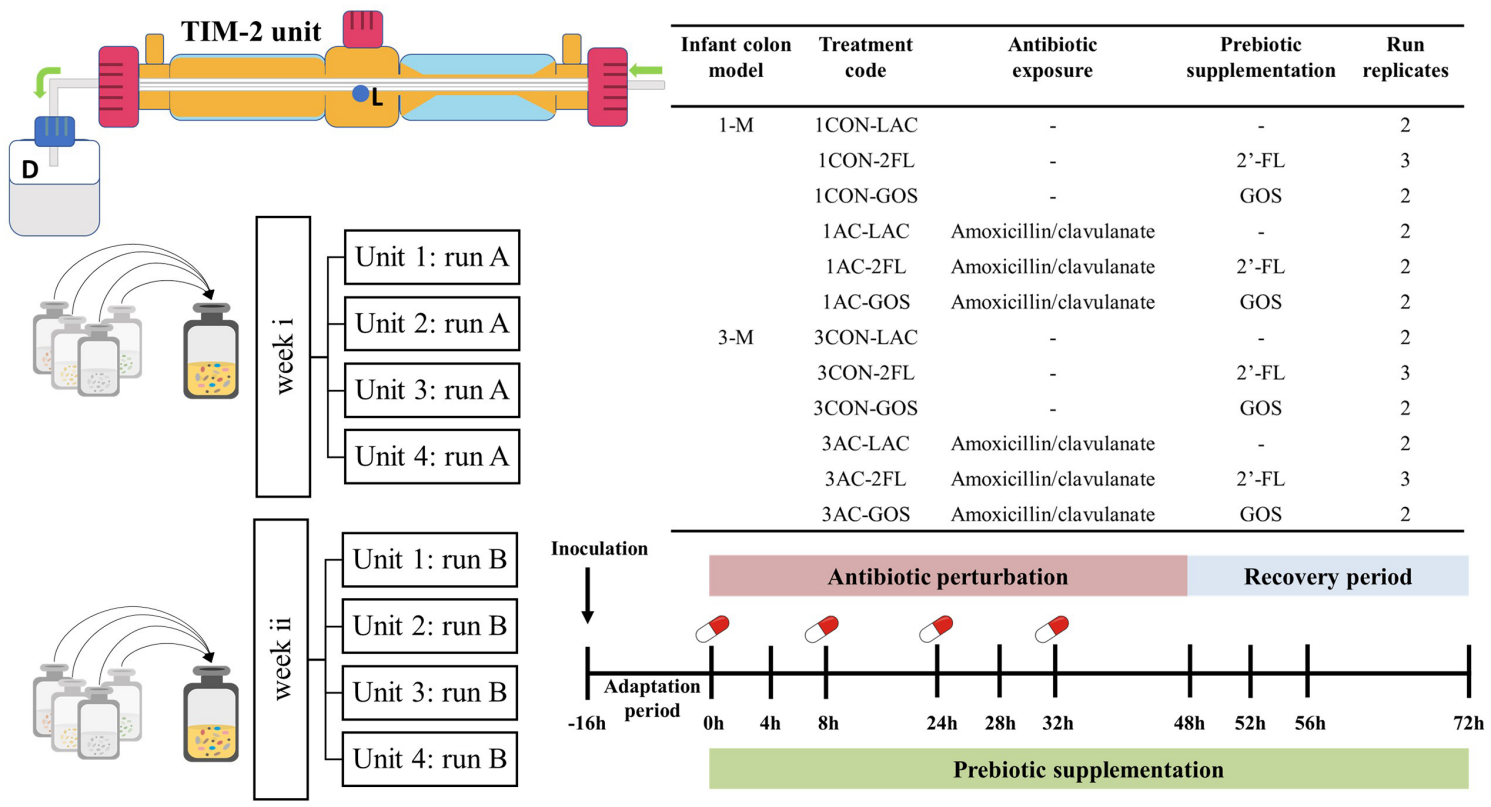
Experimental setup and sampling scheme of TIM-2 resilience study. Lumen compartment samples were taken from sampling port (L) and the dialysis liquid samples were collected from spent dialysis bottle (D). A control run without antibiotic and without prebiotics was also included as a comparison. Each treatment was run in duplicate or triplicate. Four units of TIM-2 were run in parallel.

Frozen infant fecal slurries were thawed in a water bath at 37°C for 30 min and were opened inside an anaerobic chamber (80% N_2_, 10% CO_2_, and 10% H_2_). The lumen compartment was filled with 90 mL dialysis liquid, and the TIM-2 unit was flushed with nitrogen for at least 3 h prior to inoculation with 30 mL of pooled infant fecal slurries (final concentration of 1.25% w/v). Four identical independent TIM-2 units can be run in parallel. Figure 1 shows the setup and sampling scheme of the TIM-2 experiment. Each different code of a run represents a different batch of inoculum used in the TIM-2 colon unit. The temperature was maintained at 37°C, and the pH was controlled between 6.0 and 6.2 by addition of 2 M NaOH. The i-SIEM was added in the TIM-2 model at a rate of 2.5 mL/h. The fecal microbiota was allowed to adapt to the gut model for 16 h without additional prebiotic or antibiotic.

After the adaptation period (defined as time point 0), i-SIEM supplemented with or without prebiotics was administered over a 72 h period. A pulse of antibiotic solution was administered at time points 0, 8, 24, and 32 h with a final concentration in the model of 25 μg/mL. The estimated colon concentration of antibiotic was calculated based on the typical daily dose of 30 mg amoxicillin/clavulanate per kilogram of body weight (average infant weight of 5 kg), daily food consumption and an absorption rate of 85% in the gastrointestinal tract of children (Le Blay et al., 2009). Samples were taken from the lumen compartment and dialysate collection bottle at time points − 16, 0, 4, 8, 24, 28, 32, 48, 52, 56, and 72 h. The samples at time points 0, 8, 24, and 32 h were collected just prior to antibiotic addition. The lumen compartment samples were centrifuged for 5 min at 21,000 g to separate the liquid fraction from the microbial pellet. All samples were snap-frozen in liquid nitrogen and stored at −80°C until analysis.

### DNA extraction from TIM-2 luminal samples

The pellet fraction of the luminal samples was re-suspended in 350 μL Stool Transport and Recovery (STAR) buffer (Roche Diagnostics, Indianapolis, IN, United States), then transferred to a sterile screw cap tube containing 0.25 g of 0.1 mm zirconia beads and three glass beads (diameter 2.7 mm). The samples were then subjected to a repeated bead beating procedure (three times of 5.5 m/s × 60 s) using the FastPrep-24™ 5G instrument (MP Biomedicals, the Netherlands; Salonen et al., 2010). The automated purification step was performed using Maxwell® 16Tissue LEV Total RNA purification Kit Cartridge customized for DNA purification (XAS1220) on the Maxwell® 16 Instrument (Promega, The Netherlands). Purified DNA was eluted in 35 μL nuclease-free water. Total DNA was measured using DeNovix DS-11 spectrophotometer (DeNovix Inc., Wilmington, DE, United States).

### Microbiota analysis

The V4 region of the 16S rRNA gene was amplified in duplicate using barcoded 515F (Parada et al., 2016) −806R (Apprill et al., 2015) primers. Each PCR reaction contained 10 μL of 5x Phusion Green HF buffer (Thermo Scientific), 1 μL of 10 mM dNTPs (Promega, Madison, WI, United States), 0.5 μL of Phusion Hot start II DNA polymerase (2 U/μL; Thermo Scientific), 1 μL of barcoded primers (10 μM of forward and reverse mixture), 1 μL of DNA template (20 ng/μL), and 36.5 μL nuclease-free water (Qiagen, Hilden, Germany). The PCR program consisted of initial denaturation for 30 s at 98°C, followed by 25 cycles of 98°C for 10 s, 50°C for 10 s, and 70°C for 10 s, with a final extension of 7 min at 70°C. No-template controls were included for each PCR run, and these controls gave no PCR products when visualized on agarose gels.

Subsequently, duplicate PCR products were pooled for each sample, and purified with the CleanPCR kit (CleanNA, Waddinxveen, the Netherlands). The DNA concentration was measured using QubitTM dsDNA BR Assay kit (Invitrogen by Thermo Fisher Scientific) and a DeNovix DS-11 Fluorometer (DeNovix Inc., Wilmington, DE, United States). Two mock communities of known composition and one no-template control were included for each library. An equimolar mix of purified PCR products was prepared and sent for sequencing to Novogene (Novogene-Europe, United Kingdom). The raw sequence data were processed using NG-Tax 2.0 with default settings (Poncheewin et al., 2020). Taxonomic assignment of each amplicon sequence variant (ASV) was performed based on SILVA database version 138.1 (Quast et al., 2012).

### qPCR-based total bacterial enumeration

Total bacteria 16S rRNA gene copies were quantified by qPCR using a CFX384 Touch™ Real-Time PCR Detection System (Bio-Rad, Hercules, CA, United States). As a primer-pair, we used BACT1369F/PROK1492R (Suzuki et al., 2000). The 10 μL reaction contained 5 μL iQ SYBR® Green Supermix (Bio-Rad), 0.1 μL forward primer (10 μM), 0.1 μL reverse primer (10 μM), 3.8 μL nuclease-free water, and 1 μL DNA template. The PCR amplification program consisted of pre-denaturation at 95°C for 10 min, followed by 40 cycles of denaturation at 95°C for 15 s, annealing at 60°C for 30 s, and elongation at 72°C for 15 s, ending with 95°C for 1 min and 60°C for 1 min prior to melting curve analysis from 60 to 95°C with 0.5°C temperature increment for a hold time of 5 s. The standard curves ranging from 10^1^ to 10^8^ copies of full-length 16S rRNA gene amplicons of mixed fecal bacteria were used in each run. All qPCR assays were performed in triplicate. The non-template controls did not show detectable amplification. Data were analyzed using the CFX Maestro software (Bio-Rad).

### Metabolite analysis

To remove protein from the samples, a Carrez clarification step was performed on both liquid fraction samples collected from the lumen compartment and the spent dialysis bottle of TIM-2, based on the method described by Selak et al. (2016). Briefly, the samples were diluted twice in an equal volume of 0.1 M Carrez A reagent containing K_4_Fe(CN)_6_.3H_2_O (Merck) and 0.2 M Carrez B reagent containing ZnSO_4_.7H_2_O (VWR International). The mixture was centrifuged for 5 min at 21000 g and the clear supernatant was further used for the analysis.

The concentration of 1,2-propanediol, propanol, and organic acids, including short-chain fatty acids (SCFA; acetate, propionate, butyrate, and valerate), branched-chain fatty acids (BCFA; iso-butyrate and iso-valerate), succinate, and lactate, was determined by High-Performance Liquid Chromatography (HPLC; Shimadzu LC-2030C Plus, Shimadzu Europa GmbH, Duisburg, Germany), equipped with a SUGAR SH1821 column (SHODEX, Showa Denko, Tokyo, Japan) and a refractive index detector (RID-20A, Shimadzu Europa GmbH) with a cell temperature of 40°C. The column was operated at 45°C with a flow rate of 1 mL/min, using 0.01 H_2_SO_4_ as eluent. The autosampler mixed 10 μL of external standard or collected supernatant with 10 μL of 0.01 N H_2_SO_4_, and 10 μL of this mixture was injected for analysis. The data were processed using Chromeleon™ CDS software version 7 (Thermo Fisher Scientific). The salicylate-hypochlorite method was used to measure the ammonia level in lumen and dialysate samples, based on the method described by Bower and Holm-Hansen (1980), with modification. Briefly, liquid fraction samples were diluted with Mili-Q water and 20 μL of the diluted fraction was added transferred to 96-well microplate (Greiner Bio-One). A blank sample (Milli-Q water) and a series of diluted ammonium chloride solutions (0–100 μM), were also included in the microplate. Ninety microliters of alkaline-hypochlorite solution (5% sodium hypochlorite solution was diluted 1:50 in 1.5 M sodium hydroxide solution) was added to each well and mixed by pipetting. Afterward, 90 μL salicylate solution (0.42 M sodium salicylate, 0.19 M trisodium citrate dehydrate, 0.18 M sodium potassium tartrate tetrahydrate, and 0.84 mM sodium nitroprusside) was added to each well of the plate and mixed by pipetting. The microplate was covered in aluminum foil to limit the exposure of samples to light and the mixture was incubated for 50 min at room temperature. The absorbance of samples was read at 650 nm using BioTek Epoch 2 microplate spectrophotometer (Agilent, Santa Clara, CA, United States). The concentrations were calculated based on a calibration curve of known ammonia concentrations.

Absolute quantities of each metabolite in the lumen and dialysate compartments of the TIM-2 model were calculated by multiplying the concentration in each compartment with the known or measured volume, respectively. At time 0 (T0), the concentration was artificially set at zero, and the cumulative production of the metabolites after that was calculated.

### Data analysis

Data visualization and analysis were performed in R, version 4.2.0. The absolute abundance of microbial taxa was calculated by multiplying the qPCR count of total 16S rRNA gene copies with the relative abundance of taxa, following the approach of quantitative microbiota profiling or QMP (Jian et al., 2020). Microbiota composition, at genus and ASV level, was visualized using the microViz package version 0.10.8 (Barnett et al., 2021). Taxa with unidentified genus were renamed as unidentified and followed by the lowest classifiable rank, e.g., the genus of unidentified*_Enterobacteriaceae*. ASVs were renamed to consist of genus name (or the lowest classifiable rank) and a numeric rank based on taxa abundance, e.g., the ASV of *Bifidobacterium_01* (the most abundant ASV within *Bifidobacterium* genus). To explore associations between antibiotic exposure and the absolute abundances of individual microbial taxa, a linear regression model was used to model log2-transformed absolute abundance of each microbial taxon per time point and the resulting coefficients were visualized using the microViz package.

The non-phylogenetic diversity estimates were generated using the microbiome package version 1.19.0 (Lahti and Shetty, 2017), including observed genera and Shannon diversity, in which the exponential of Shannon index was further calculated as Shannon effective number of genera. The phylogenetic diversity was calculated using the picante package version 1.8.2 (Kembel et al., 2010). The alpha diversity was visualized in box plots using the ggpubr package version 0.4.0 (Kassambara and Kassambara, 2020). The Wilcoxon test was performed to test the differences in alpha diversity between the groups treated with and without antibiotic using the rstatix package version 0.7.0 (Kassambara, 2021). In order to visualize the variation in microbiota composition between samples at different time points, principal component analysis (PCA) and principal coordinate analysis (PCoA) were used on log10-transformed absolute abundances of genera and GUniFrac distances, respectively. Principal Response Curve (PRC) analysis was performed on log10-transformed absolute abundances of genera using the vegan package version 2.6.2 (Oksanen et al., 2022). PRC analysis on GUniFrac distances was conducted using the distance-based PRC (dbPRC) approach (Shankar et al., 2017). PRC and dbPRC analyses were performed by comparing the control group (without antibiotic and without prebiotic) to antibiotic-treated groups with and without prebiotic (2’-FL or GOS).

The metabolite data were calculated as cumulative production for metabolites detected in both luminal compartment and spent dialysis liquid, or otherwise presented as detection in luminal compartment only in case of the absence of the metabolites in the dialysis liquid. To explore associations between antibiotic or prebiotic treatment and the production of each metabolite, a linear regression model was used to model log2-transformed concentration of each metabolite per time points and the resulting coefficients were visualized using microViz package. The graphs of metabolite amounts from each treatment run over time were generated using the ggplot2 package version 3.3.6 (Wickham, 2016). Spearman correlation test between rate of metabolite production and taxa was performed following the script provided in the Rhea pipeline (Lagkouvardos et al., 2017). For correlation analyses, the rate of metabolite production was calculated by dividing the change in total metabolite amount (mmol) by the change in time (hours) since the previous measurement time point. For metabolites that were never detected in the dialysis liquid, the total amount is equal to the amount in the lumen. Correlation network was constructed using visNetwork package version 2.1.1 (Almende, 2022), visualizing significant correlations with FDR corrected *p* value <0.05 and correlation coefficient > 0.5.

## Results

### Amoxicillin/clavulanate induced changes in microbiota composition

**Figure 2:**
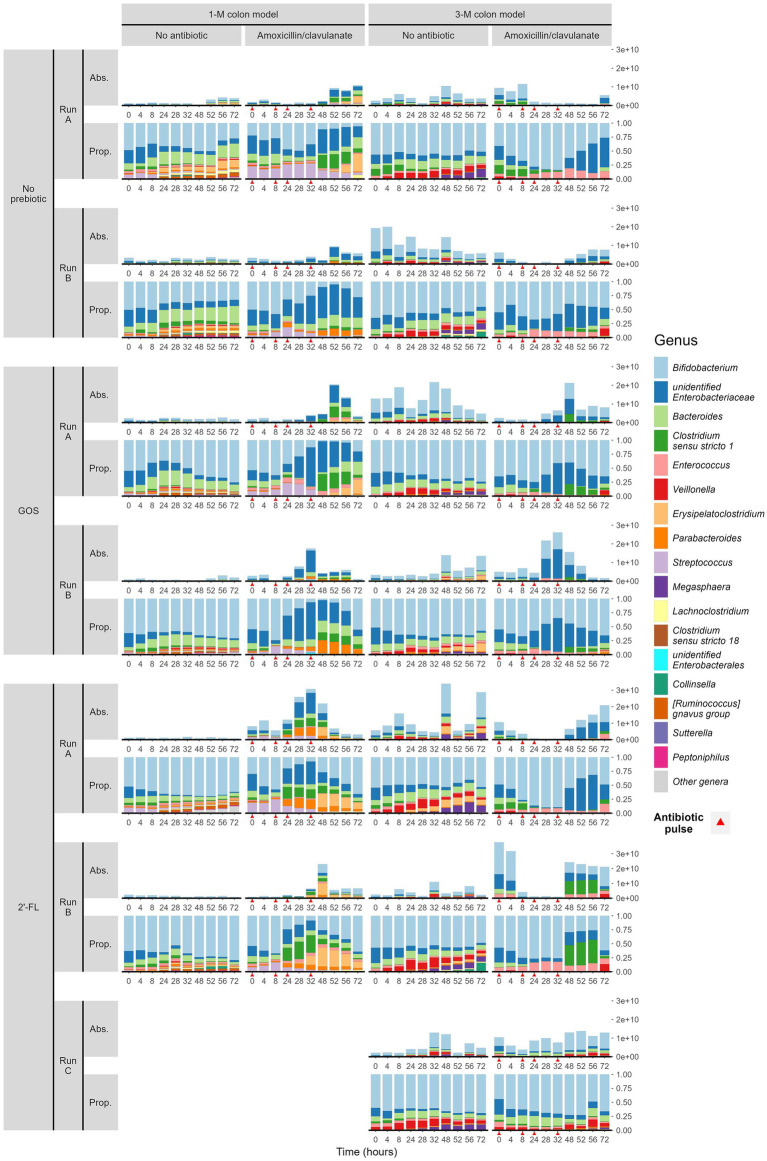
Microbial composition (absolute abundance and proportion) at genus level of infant gut microbiota cultivated in TIM-2. Samples were grouped by the simulated age group of the colon model (1- or 3-M) and treatment (with or without antibiotic, and with or without either prebiotic, GOS, or 2’-FL). Each treatment was performed in duplicate or triplicate, with each run labeled A, B, or C. Antibiotics were added at 0, 8, 24, and 32 h, immediately after sampling (indicated by red arrows).

**Figure 3:**
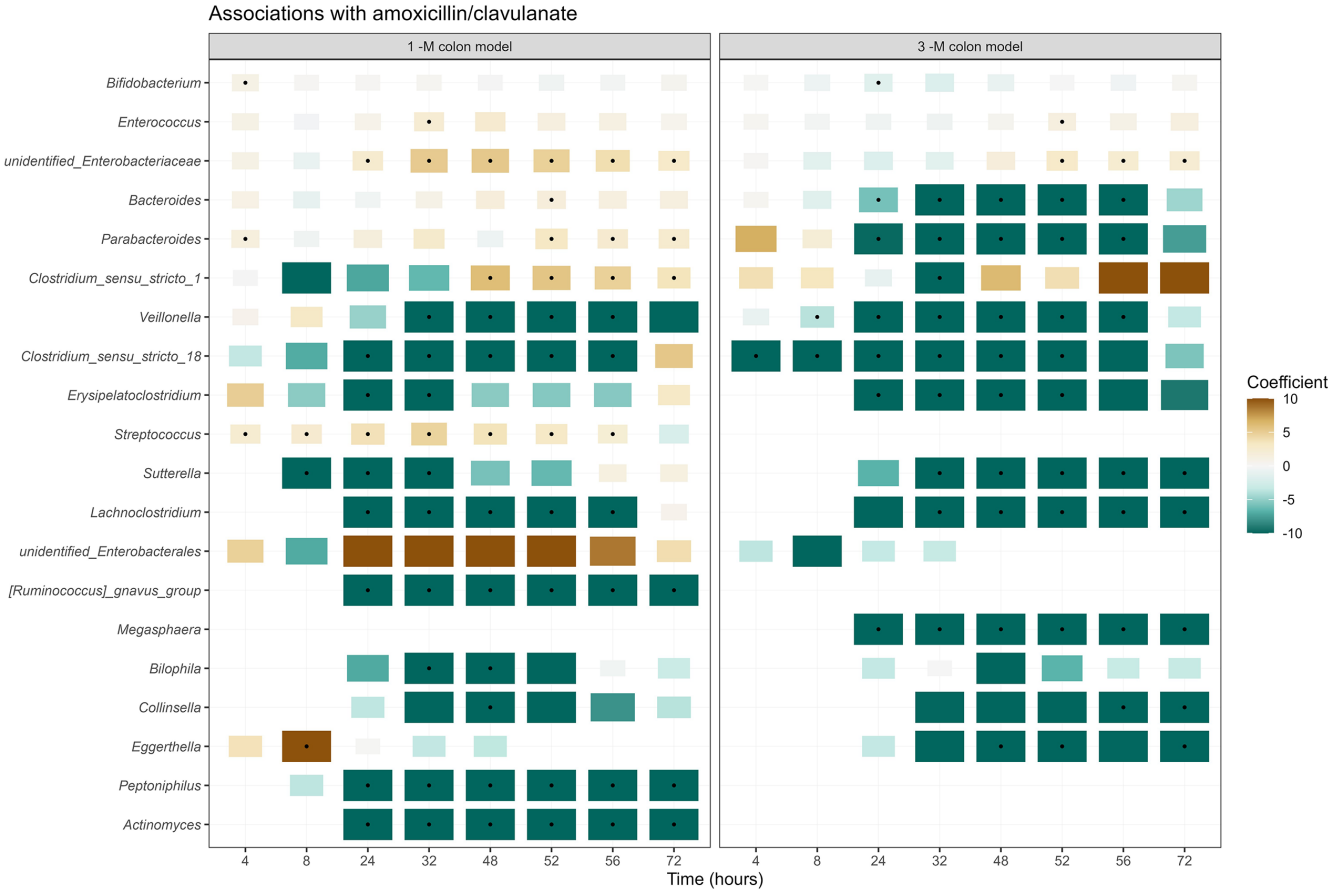
Taxa associated with exposure to amoxicillin/clavulanate. A summary of the results of linear regression models of log2-transformed absolute abundances per time point, per taxon, are visualized in the heatmap. The black dots indicate statistically significant associations (*p* < 0.05). The color of each tile represents the coefficient for the association between each genus and the exposure to antibiotic. The size of the tile is proportional to the absolute coefficient value.

The use of amoxicillin/clavulanate induced changes in the composition of infant gut microbiota in the *in vitro* gut model. Compared to the group without antibiotic in the 1-M colon model (1CON-LAC, 1CON-2FL, and 1CON-GOS), the total bacterial 16S rRNA gene counts were significantly higher in the antibiotic-treated group (1 AC-LAC, 1 AC-2FL, and 1 AC-GOS) at time point 52 and 56 h (Supplementary Figure 2). In both 1-M and 3-M colon models, antibiotic-treated microbiota shifted to a community associated with a higher relative abundance of *Clostridium sensu stricto* 1 and unidentified genus within the *Enterobacteriaceae* family (Figure 2). The exposure of amoxicillin/clavulanate was associated with a decreased absolute abundance of the majority of taxa (Figure 3). Moreover, the absolute abundance of unidentified genus of *Enterobacteriaceae*, *Parabacteroides*, *Clostridium sensu stricto* 1, and *Streptococcus* was significantly higher in the antibiotic-treated microbiota of the 1-M colon model compared to the group without antibiotic at multiple time points. In the antibiotic-treated microbiota of the 3-M colon model, compared to the untreated group, the absolute abundance of unidentified genus of *Enterobacteriaceae* increased while a decrease was seen in the absolute abundance of *Bacteroides*, *Parabacteroides*, and *Megasphaera*, among other taxa. In the 1-M colon model, the antibiotic-treated microbiota dominated by unidentified *Enterobacteriaceae* 02 ASV (in both runs of 1 AC-2FL and one run of 1 AC-LAC or 1 AC-GOS) showed a relatively faster recovery at time point 72 h compared to the community associated with unidentified *Enterobacteriaceae* 01 ASV (Supplementary Figure 3).

In the absence of amoxicillin/clavulanate, the supplementation of GOS and 2’-FL in the 1-M colon model promoted an increase in the relative abundance of *Bifidobacterium*, compared to the control group without any prebiotics (Figure 2). In the 1-M colon model, the supplementation of 2’-FL consistently promoted the shift of antibiotic-treated microbiota to a community associated with a higher relative abundance of *Bifidobacterium* from time point 48 h. In the 3-M colon model, the supplementation of GOS promoted the shift of antibiotic-treated microbiota to a community associated with a higher relative abundance of *Bifidobacterium* at time point 52 h.

### Amoxicillin/clavulanate reduced alpha diversity

**Figure 4:**
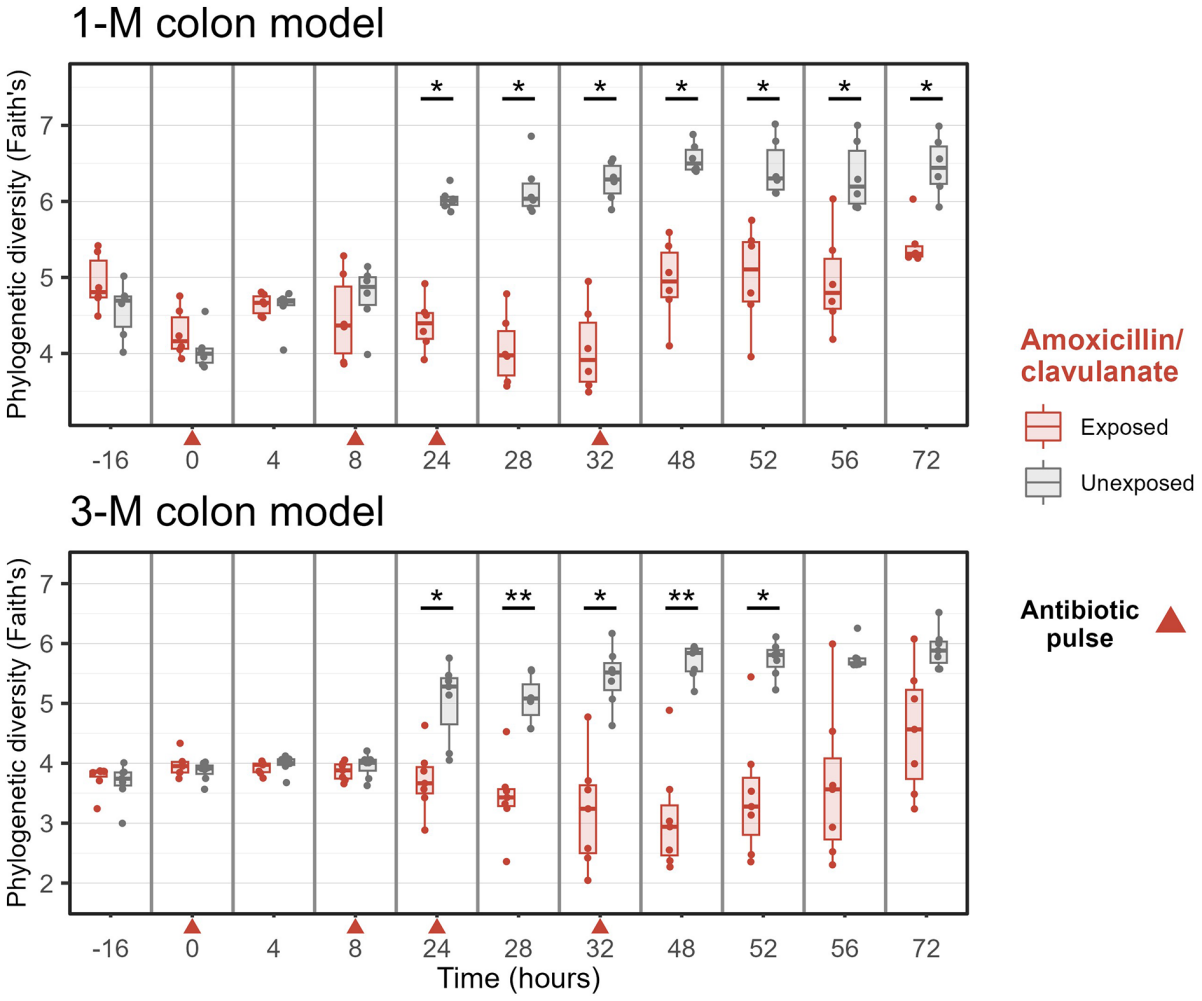
Phylogenetic diversity of microbiota of groups treated with and without amoxicillin/clavulanate. Antibiotics were added at 0, 8, 24, and 32 h, immediately after sampling (indicated by red arrows). Wilcoxon tests were used to compare the diversity with and without antibiotic. Significant differences are indicated by ^*^p < 0.05 and ^**^p < 0.01.

Furthermore, the exposure to amoxicillin/clavulanate decreased the phylogenetic diversity of microbiota in 1-M and 3-M colon models from time point 24 h (Figure 4). After the last exposure to the antibiotic, the phylogenetic diversity slowly recovered, but did not reach the values of the non-treated samples at 72 h and was still statistically significantly different in 1-M microbiota. Moreover, the phylogenetic diversity of the 3-M microbiota was not yet completely recovered at 72 h, although the differences were only statistically significant up to 52 h. The same pattern was seen in the observed number of genera between the groups with and without antibiotic exposure for either of the age groups, while a significant difference in effective Shannon diversity of genera (the number of equally abundant genera) between the antibiotic-treated group compared to the untreated group was only observed at time point 8–48 h in the 3-M colon model (Supplementary Figure 4).

### Amoxicillin/clavulanate induced time-dependent deviations in microbiota composition

**Figure 5:**
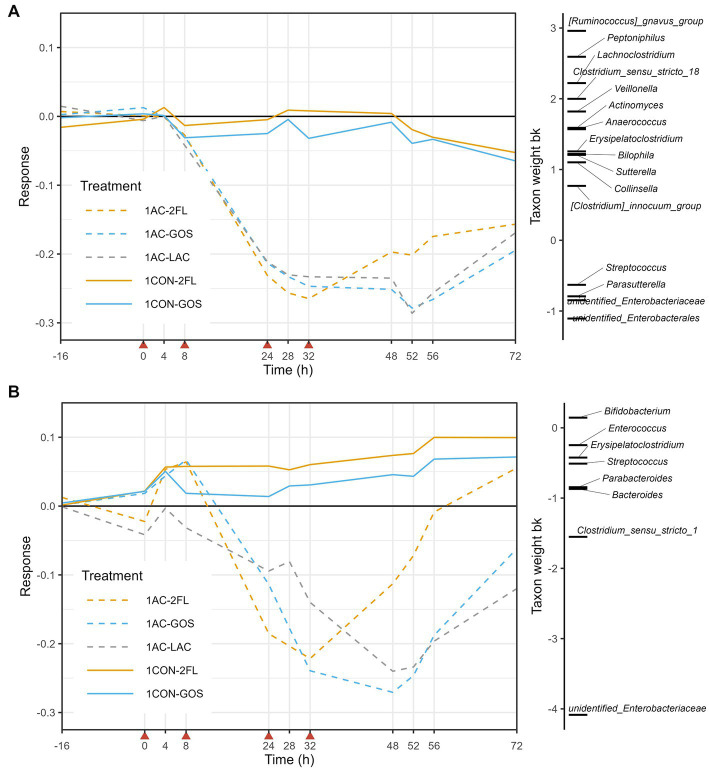
Principal response curve (PRC), resulting from the analysis of microbiota grouped by treatment in the 1-M colon model. The analysis was performed on log10-transformed absolute abundances of genera **(A)** and GUniFrac distance **(B)**. The affinity of a taxon to the PRC is shown as taxon weight (bk). Only taxa with bk values above 0.5 or below −0.5 are displayed. Antibiotics were added at 0, 8, 24, and 32 h, immediately after sampling (indicated by red arrows).

Ordination visualizations suggest that amoxicillin/clavulanate exposure initially caused disturbance in overall microbiota composition within the TIM-2 model, with clear separation seen on PCA on log10-transformed absolute abundance (Supplementary Figure 5) and GUnifrac-PCoA (Supplementary Figure 6) at time point 24 h, for both 1- and 3-M models. In order to evaluate changes in microbiota composition due to time-dependent treatment effects, Principal Response Curve (PRC) analysis was performed in which the trajectory of the control runs without antibiotic and prebiotic were used as a reference (1CON-LAC and 3CON-LAC in 1-M and 3-M colon models, respectively). In 1-M microbiota, the PRC on log10-transformed genus-level absolute abundances estimated that time explained 36% of the variance while the treatment and its interaction with time explained 48%. The microbiota of amoxicillin/clavulanate treated group (1 AC-LAC, 1 AC-2FL, and 1 AC-GOS) started to deviate from the reference (1CON-LAC) and prebiotic supplemented group (1CON-2FL and 1CON-GOS) before time point 24 h (no sample for microbiota analysis was taken between antibiotic addition at 8 and 24 h) and started to recover at time point 48 h for 1 AC-2FL treatment and at time point 56 h for both 1 AC-LAC and 1 AC-GOS (Figure 5A).

The taxon weight axis shows the affinity of each taxon (at genus level) to the treatment response shown in the PRC diagram. The sign of the taxon weight indicates the direction of the changes, while the score magnitude reflects the size of the changes. The positive weighting of a taxa group indicate a decrease in their absolute abundance due to antibiotic treatment, including *Ruminococcus gnavus* group, *Lachnoclostridium*, *Peptoniphilus*, *Clostridium sensu stricto* 18, and *Veillonella*, among others. In contrast, the negative weighting of a taxa group indicate an increase in their absolute abundance due to antibiotic treatment, including unidentified genus of *Enterobacteriaceae*, unidentified genus of *Enterobacterales*, *Streptococcus*, and *Parasutterella*. These changes in the absolute abundances (QMP) of taxa listed in the PRC of 1-M microbiota can be further seen in Supplementary Figure 7. Moreover, GUniFrac distance-based PRC analysis on the first axis showed that time explained 32% of the variance while the interaction between time and treatment with antibiotic explained 59%. In this GUniFrac-PRC, unidentified genus of *Enterobacteriaceae* and *Clostridium sensu stricto* 1 were the main drivers of microbiota changes due to antibiotic treatment, while supplementation of 2’-FL (1 AC-2FL) supported full recovery from time point 56 h (Figure 5B).

**Figure 6:**
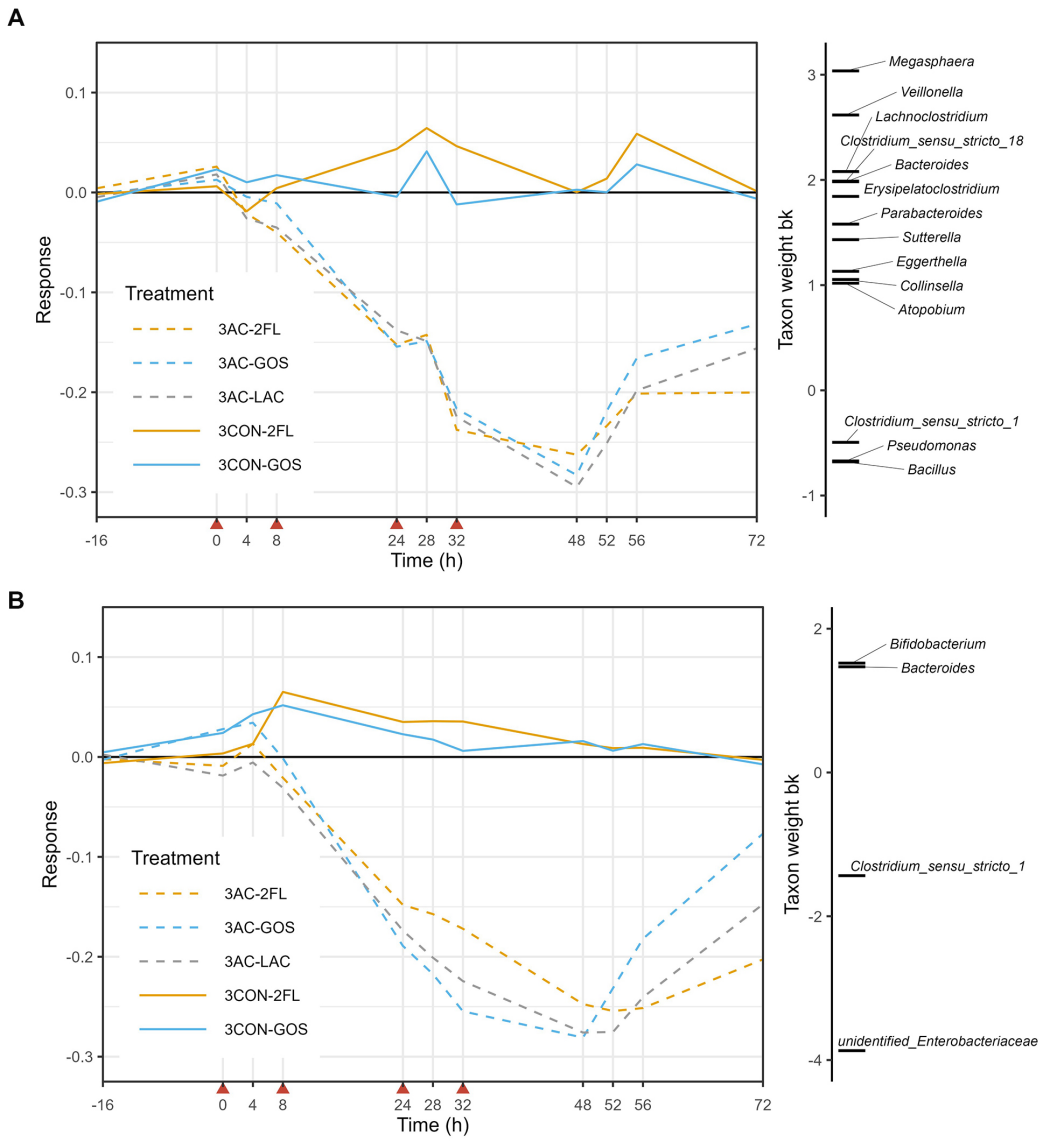
Principal response curve (PRC), resulting from the analysis of microbiota grouped by treatment in the 3-M colon model. The analysis was performed on log10-transformed relative abundances of genera **(A)** and GUniFrac distance **(B)**. The affinity of a taxon to the PRC is shown as taxon weight (bk). Only taxa with bk values above 0.5 or below −0.5 are displayed. Antibiotics were added at 0, 8, 24, and 32 h, immediately after sampling (indicated by red arrows).

In the case of 3-M microbiota, PRC analysis based on log10-transformed absolute abundances indicated that time explained 27% of the variance, while the interaction between treatment and time explained 46%. The antibiotic-treated microbiota (3 AC-LAC, 3 AC-2FL, and 3 AC-GOS) started to deviate before time point 24 h and start of recovery was seen from time point 52 h (Figure 6A). The positively weighted taxa groups indicating a decrease in their absolute abundance due to antibiotic treatment, included *Megasphaera*, *Veillonella*, *Lachnoclostridium*, *Clostridium sensu stricto* 18, and *Bacteroides*, among others. In contrast, the negatively weighted taxa groups indicating an increase in their absolute abundance, included *Clostridium sensu stricto* 1, *Bacillus*, and *Pseudomonas*. These changes in the absolute abundances (QMP) of taxa listed in the PRC of 3-M microbiota can be further seen in Supplementary Figure 8. GUniFrac-PRC, which incorporated phylogenetic information, showed that time explained 29% of the variance while the interaction between time and treatment with antibiotic explained 55%. In this PRC, we observed that unidentified genus of *Enterobacteriaceae*, *Clostridium sensu stricto* 1, *Bacteroides*, and *Bifidobacterium* were the main driver of the microbiota changes due to antibiotic, with supplementation of GOS on antibiotic-treated microbiota (3 AC-GOS) having the least deviation from controls at time point 72 h (Figure 6B).

### Antibiotic and prebiotics influenced microbiota activity

**Figure 7:**
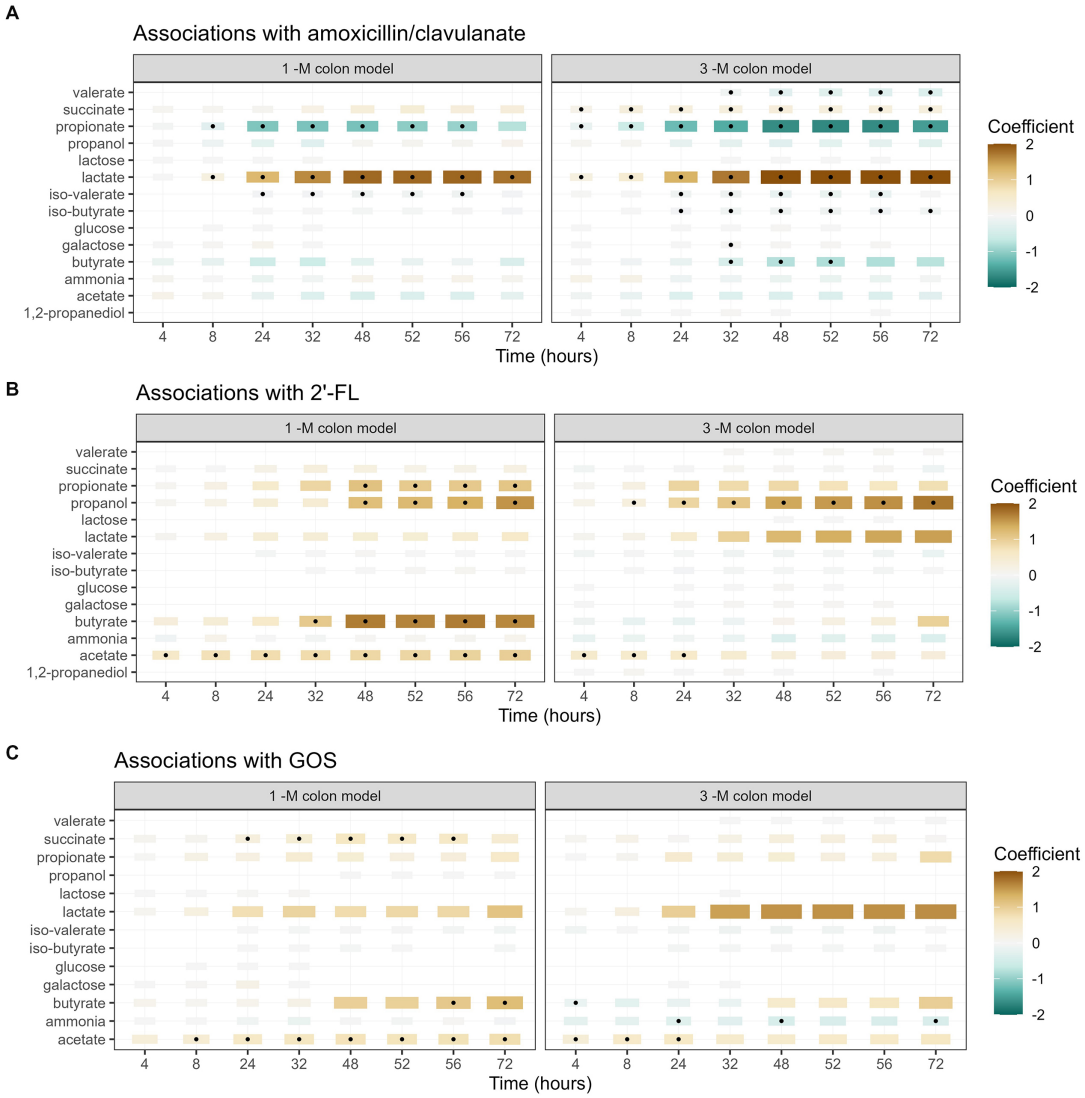
Associations between each metabolite and the addition of amoxicillin/clavulanate **(A)**, 2’-FL **(B)**, and GOS **(C)**. A summary of the results of linear regression models of log2-transformed metabolite concentration per time point, per metabolite, are visualized in the heatmap. The black dots indicate statistically significant associations (*p* < 0.05). The color of each tile represents the coefficient for the association between each metabolite and each exposure at that time point. The size of the tiles is proportional to absolute coefficient.

The exposure to amoxicillin/clavulanate also affected the activity of the microbiota, as evaluated based on the production of microbial metabolites, including SCFA, BCFA, ammonia, and other metabolites. Antibiotic exposure showed significant associations with an increased lactate production and a decrease in the production of propionate and iso-valerate in the 1-M model (Figure 7A). In the 3-M colon model, the exposure of amoxicillin/clavulanate showed a significant association with a higher production of succinate and lactate and a lower production of propionate, butyrate, and valerate, along with a lower level of iso-butyrate and iso-valerate.

The supplementation of 2’-FL was significantly associated with a higher production of acetate and propanol in both 1-M and 3-M colon models, compared to the treatment lacking of prebiotics (Figure 7B). In addition, the supplementation of 2’-FL was associated with a higher production of propionate and butyrate at multiple time points in the 1-M colon model. The supplementation of GOS was significantly associated with a higher production of acetate, butyrate and succinate in the 1-M colon model, compared to the treatment lacking of prebiotics (Figure 7C). In the 3-M colon model, the supplementation of GOS was weakly associated with a higher production of acetate and a lower production of ammonia at multiple time points. In addition, an increasing trend was seen in butyrate production from both runs of antibiotic-treated microbiota supplemented with GOS (3 AC-GOS), compared to the lack of butyrate produced in antibiotic-treated microbiota without prebiotic (Supplementary Figure 9).

It is worth noting that some traces of lactose, galactose, and glucose were detected at a specific time point during the antibiotic course in multiple runs of 1 AC-GOS (Supplementary Figure 9). Moreover, we detected some traces of lactose, glucose, and galactose at the time points between 28 and 72 h in one of the runs in the antibiotic-treated group supplemented prebiotics (3 AC-2FL and 3 AC-GOS). In both 1-M and 3-M models, some traces of GOS were also detected both in the luminal compartment and dialysis liquid of the antibiotic-treated group at time point 28 and 32 h, indicating incomplete degradation (Supplementary Figure 10).

### Amoxicillin/clavulanate shifted bacteria-metabolites correlation network

**Figure 8:**
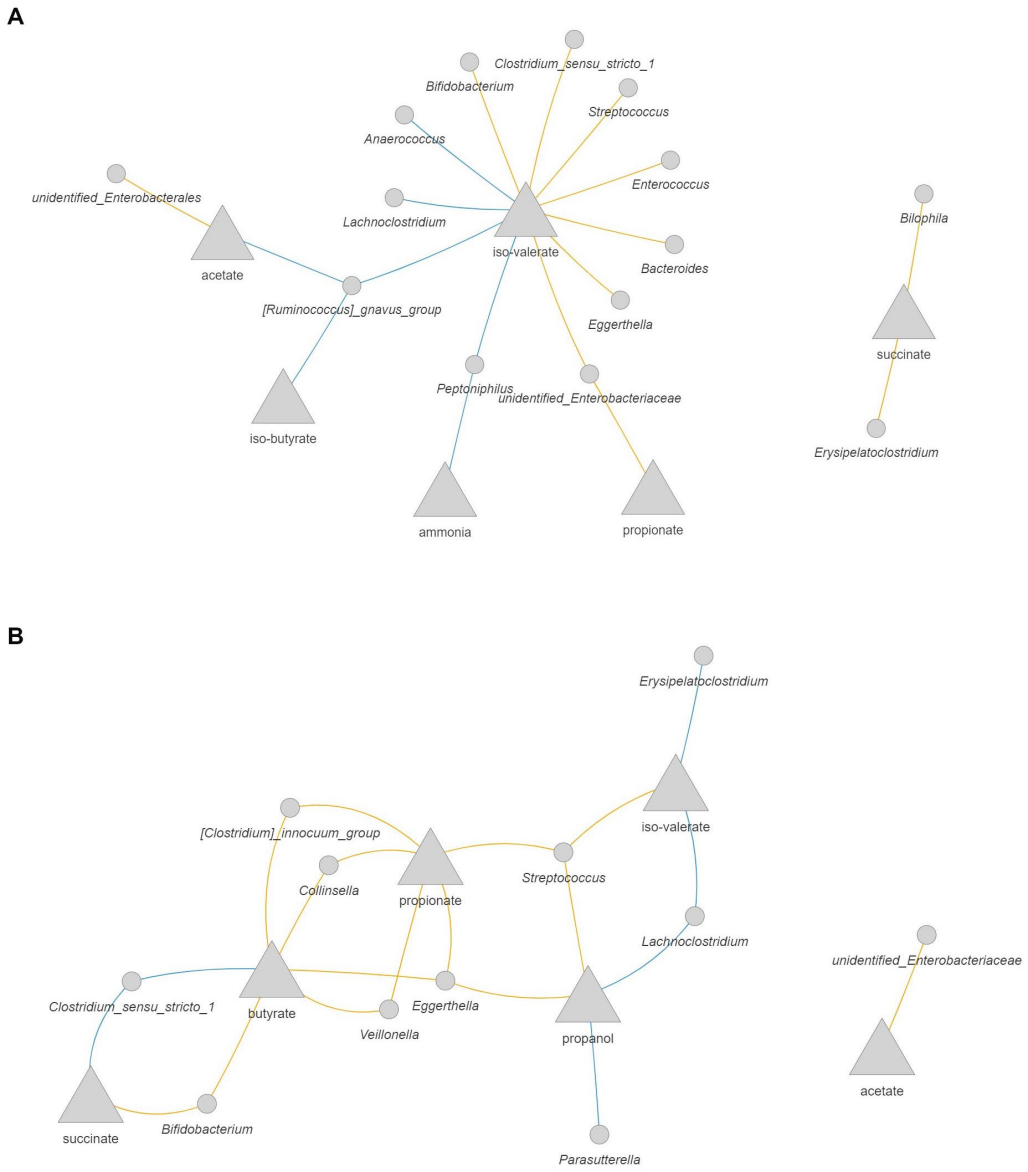
Correlation network between microbial metabolites and bacteria in the 1-M colon model without antibiotic **(A)** and with antibiotic **(B)**. The metabolites are presented as the rate of production by dividing the delta production by the delta time. Only significant correlations (FDR corrected *p* < 0.05) with coefficient larger than 0.5 are shown. Blue: positive correlation; orange: negative correlation.

As changes in the composition of the microbiota might affect its activity, we evaluated possible correlations between microbial metabolites and the abundance of bacterial taxa from the quantitative microbial profiling of 16S rRNA genes. In the 1-M colon model without antibiotic (Figure 8A) larger interconnected networks were seen for the production of iso-valerate, including positive correlation of this BCFA with mucin degrading bacteria (*Peptoniphilus*, *Ruminococcus gnavus* group, and *Lachnoclostridium*). Moreover, the abundance of *Peptoniphilus* was positively correlated with ammonia and the abundance of *Ruminococcus gnavus* group was positively correlated with iso-butyrate and acetate production. For the antibiotic-exposed microbiota, the level of iso-valerate was positively correlated with *Lachnoclostridium* and *Erysipelatoclostridium* (Figure 8B). An increase in succinate and butyrate production was correlated with an increase in the abundance of *Clostridium sensu stricto 1*, while these metabolites were negatively correlated with *Bifidobacterium*. The production rate of propanol was positively correlated with the abundance of *Parasutterella* and *Lachnoclostridium*.

**Figure 9:**
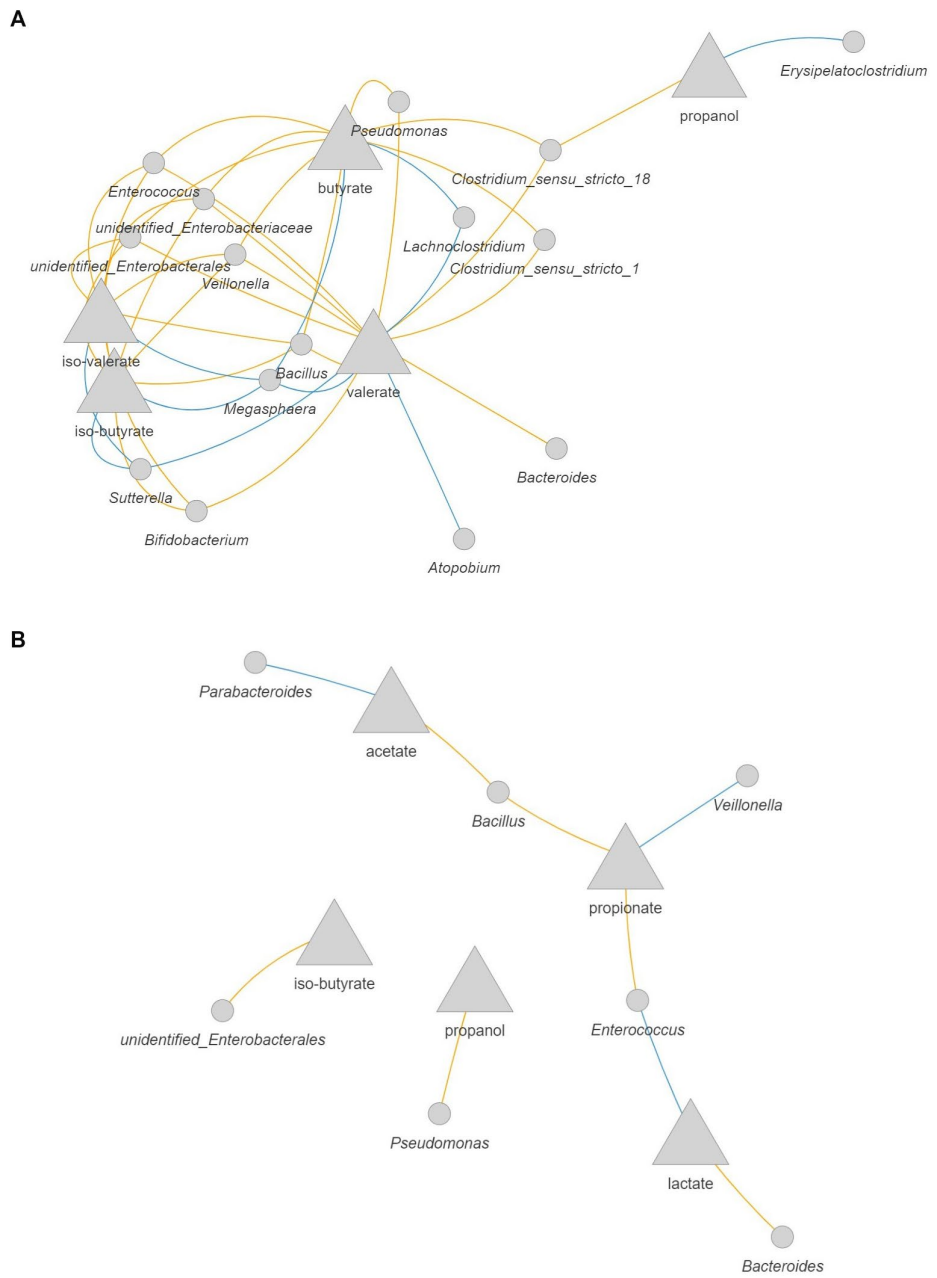
Correlation network between microbial metabolites and bacteria in the 3-M colon model without antibiotic **(A)** and with antibiotic **(B)**. The metabolites are presented as the rate of production by dividing the delta production by the delta time. Only significant correlations (FDR corrected *p* < 0.05) with coefficient larger than 0.5 are shown. Blue: positive correlation; orange: negative correlation.

In the 3-M colon model without antibiotic, intermingled networks were displayed for the production rate of valerate, iso-butyrate, and iso-valerate, including a positive correlation between these metabolites and the abundance of *Megasphaera* and *Sutterella* (Figure 9A). With respect to valerate, a positive correlation was also found with increased abundance of *Atopobium* and *Lachnoclostridium*. In addition, the increase in the production rate of butyrate was positively correlated with the increase in the abundance of *Lachnoclostridium* and *Megasphaera*, which are known as butyrate-producing bacteria. The production rate of propanol was positively correlated with *Erysipelatoclostridium*. In the antibiotic-exposed 3-M colon model, the production rate of lactate was positively correlated with the abundance of *Enterococcus*, while this intermediate was negatively correlated with the abundance of *Bacteroides* (Figure 9B). The increased production rate of propionate was correlated with increased abundance of *Veillonella* and the production rate of acetate was correlated positively with the abundance of *Parabacteroides*, while this SCFA was negatively correlated with the abundance of *Bacillus*.

## Discussion

In the experimental setup used in this study, the dynamics of a community structure of mixed bacteria was simulated in the 1-M colon model, whereas the dynamics of a *Bifidobacterium* dominated community (relative abundance >50%) was simulated in the 3-M colon model. These microbiota profiles have often been observed in feces from 1- to 3-month old infants in other studies (Borewicz et al., 2019). Our use of a dynamic *in vitro* gut model provides insight into the responses of these typical infant gut microbial ecosystems to amoxicillin/clavulanate exposure. Amoxicillin/clavulanate treatment decreased the diversity of microbiota, in accordance with a previous study on amoxicillin/clavulanate treated children (Mancabelli et al., 2021). The changes of microbiota composition due to amoxicillin/clavulanate were evidenced by variation in the relative abundance of enterobacteria, enterococci, clostridia, and bifidobacteria, next to a temporary decrease in the relative abundance of *Bacteroides* and *Veillonella*, similar to an *in vivo* study in infants treated with amoxicillin (Korpela et al., 2020).

In both age groups, the exposure to amoxicillin/clavulanate induced a temporary shift to a community dominated by enterobacteria. Some members of gut microbiota are less sensitive to amoxicillin/clavulanate due to the ability to produce β-lactamases at levels exceeding the inhibitory effects of clavulanate, including *Bacteroides* and some genera within the *Enterobacteriaceae* family, such as *Escherichia*, *Klebsiella*, and *Citrobacter* (Nakano et al., 2011; Duytschaever et al., 2013). In addition, periplasmic β-lactamases of Gram-negative bacteria offer protection to individual cells, in contrast to the extracellular β-lactamases of Gram-positive bacteria that reduce the environmental concentration of β-lactam antibiotics (Livermore, 1997). Thus, this resistance mechanism, in relation with β-lactamases quantity and location, might explain the increase in the abundance of enterobacteria during antibiotic treatment. Although most intestinal *Bifidobacterium* spp. are sensitive to amoxicillin/clavulanate, specific strains of *Bifidobacterium breve* and *Bifidobacterium longum* were less sensitive to this antibiotic, where the presence of ABC transporter-encoding genes have been implicated to play a role in this observed insensitivity (Mancabelli et al., 2021), suggesting that variation in the level of resistance to amoxicillin/clavulanate was species/strain-specific. In the case of antibiotic-producing bacteria, the presence of an ABC transporter is important for the self-resistance mechanism by transporting the antibiotic through the cell membrane and preventing the antibiotic from entering the cell (Mendez and Salas, 2001).

The use of amoxicillin/clavulanate also affected microbiota activity in both age groups, indicated by an increase of succinate and lactate production, while a decrease was seen in the production of propionate, butyrate, valerate, iso-butyrate, and iso-valerate, compared to the unexposed group. An increase in lactate was also seen in the gut microbiota of children treated with amoxicillin in another *in vitro* gut model (Le Blay et al., 2009). This increase of intermediate metabolites and the decrease of propionate might be due to the decrease in the abundance of lactate-utilizing propionate-producing bacteria, including *Peptoniphilus*, *Veillonella*, and *Megasphaera* (Louis et al., 2022). The importance of lactate-utilizing bacteria in the infant gut should not be underestimated as they play an important role in preventing accumulation of lactate and maintaining colonic health when they are in the right balance (Louis et al., 2022). On the other hand, a higher abundance of lactate-utilizing H_2_-producing bacteria, such as *Veillonella* and *Eubacterium hallii*, has been associated with infant colic, or excessive crying, as excess production of H_2_ seems to be responsible for bloating and cramping (Pham et al., 2017).

Besides the increase of intermediate metabolites, low production of butyrate might be due to a variation in the abundance of butyrate-producing bacteria, including *Peptoniphilus*, *Megasphaera*, and endospore-forming bacteria such as *Ruminococcus*, *Erysipelatoclostridium*, and *Clostridium* (Appert et al., 2020). With respect to valerate, bacteria can produce this SCFA from ethanol and propionate or from amino acid fermentation by some *Clostridium* species (Buckel, 2001; Candry et al., 2020). In addition, *Megasphaera* isolated from the human gut also showed the ability to produce valerate (Shetty et al., 2013). The exposure of amoxicillin/clavulanate also decreased the production of BCFA compared to the unexposed group, indicating that the antibiotic also affected protein fermentation to a certain extent, due to variation in BCFA producers such as *Bacteroides*, *Megasphaera*, and *Clostridium* (Allison, 1978; Smith and Macfarlane, 1998).

The influence of prebiotics on the resilience of microbiota structure and activity likely depends on the degrading capacity of the disturbed microbiota. Our data indicate that the supplementation of 2’-FL is beneficial in the recovery of the community characterized by mixed taxa in the 1-M colon model. *Bifidobacterium* and *Bacteroides* utilize 2’-FL to produce SCFA and lactate (Yu et al., 2013). In the absence of antibiotic, the fermentation of 2’-FL led to an increase in propanol and production of the SCFA acetate, propionate, and butyrate, as also seen previously in other *in vitro* gut models (Salli et al., 2019; Van den Abbeele et al., 2019, 2021; Natividad et al., 2022). In the antibiotic-exposed group, the supplementation of 2’-FL maintained the butyrate production and recuperated the production of propionate. Within bifidobacteria, the strategies for utilizing 2’-FL vary for different species and strains, with an intracellular digestion strategy by *B*. *longum* subsp. *infantis* and extracellular degradation by *B*. *bifidum* (Sakanaka et al., 2019). Fucose can be either used by *B*. *infantis* to produce 1,2-propanediol or excreted from the cell (Asakuma et al., 2011; Zabel et al., 2020). Moreover, extracellular degradation of 2’-FL by *B*. *bifidum* allows cross-feeding of fucose and galactose to other gut bacteria (Asakuma et al., 2011). Other gut bacteria are also able to utilize fucose from fucosylated sources, such as *Ruminococcus gnavus* that produce propanol and propionate when grown on 2’-FL, as demonstrated previously in an *in vitro* study (Crost et al., 2013). It is worth noting that intermediate metabolites such as 1,2-propanediol can be further utilized by gut bacteria to propanol and propionate. An increase in the detection of 1,2-propanediol in the lumen indicated that amoxicillin/clavulanate treatment affected the cross-feeding of this intermediate metabolite by inhibiting the 1,2-propanediol-utilizing bacteria, such as *Lachnoclostridium*, *Lactobacillus*, *Veillonella*, and *Blautia* (Louis and Flint, 2017).

In one run of the antibiotic-exposed 3-M colon model, some traces of glucose, galactose, and lactose were detected in the lumen of 2’-FL supplemented experiment (3 AC-2FL), specifically when the community shifted to one dominated by enterobacteria, as such a community has previously been classified as 2’-FL slow-degrading microbiota (Salli et al., 2019). Cultivating a stable community of fecal-derived microbiota remains a challenge due to their complexity, hence resulting in some variation in microbiota composition among different runs. In particular, the differences seen within treatment group 3 AC-2FL might be explained by the possibility of reaching alternative (multiple) states of the community due to antibiotic perturbation in combination with niche selection from the supplementation of 2’-FL. In addition, the response of a complex ecosystem of microbes to a perturbation is not fully deterministic as the range of dynamic responses is partly stochastic or probabilistic. Therefore, the variation in responses between each run was seen even under the same well-controlled conditions, and hence the need for replication of each condition.

In addition, an incomplete fermentation of GOS was seen in the antibiotic-treated group of both ages, indicated by the detection of glucose, galactose, and lactose in the lumen and traces of GOS in both the lumen and dialysis liquid, as previously seen *in vitro* in adult microbiota, which was strongly disturbed by amoxicillin (Ladirat et al., 2014b). To this end, our data indicate that the supplementation of GOS is beneficial for the recovery of the microbiota dominated by *Bifidobacterium* in the 3-M colon model. A lower level of monosaccharides and lactose in this group suggested that cross-feeding on GOS was still stimulated. In the presence of antibiotic, the supplementation of GOS in this age group maintained the production of acetate and butyrate, comparable to the treatment without antibiotic, as previously observed in another gut model (Salli et al., 2019). Several gut bacteria are able to utilize a broad range of GOS molecules, including *Bifidobacterium*, *Bacteroides*, and *Lactobacillus* (Böger et al., 2019b). Moreover, extracellular enzyme produced by gut bacteria, including endo-galactanase (*Bifidobacterium breve* and *Bacteroides thetaiotaomicron*) and β-galactosidase (*Bifidobacterium bifidum*), allowing degradation and utilization of GOS with higher DP, but also allowing cross-feeding with other bacteria (Böger et al., 2019a,b).

The use of a validated, dynamic *in vitro* gut model offered a well-controlled ecosystem that allowed a direct comparison between treatments. The TIM-2 colon model also provides the possibility in adding multiple pulses of antibiotic for studying the dynamic response of microbiota, which otherwise requires a difficult design to be conducted in *in vitro* batch systems. However, gas production was not measured in our study. This might give more insight into the potential overproduction of specific gasses due to antibiotic disturbance, such as H_2_. We also acknowledge the limitation in taxonomic classification below genus level (and family level for some bacterial groups), as the V4 region of 16S rRNA gene is highly similar or identical across multiple species. Furthermore, the use of individual feces might be of interest in evaluating possible individual responses to antibiotic disturbance or to prebiotic supplementation. Depending on the degree of perturbation, a prolonged fermentation period seems to be necessary to investigate a long-term impact of antibiotic treatment. To this end, the study described here used a relatively short-term antibiotic course and only one type of antibiotic as a model, thus additional research is needed to study the effect of the period and type of antibiotic on microbiota changes and the potential of prebiotics, or combinations thereof, to mitigate or restore any dysbiosis.

## Conclusion

The use of amoxicillin/clavulanate induced changes in the composition and activity of pre-weaning infant gut microbiota. The compositional changes were characterized by a temporary shift to a community dominated by enterobacteria. The supplementation of 2’-FL promoted the recovery of microbiota with mixed taxa, while GOS promoted the recovery of bifidobacteria dominated microbiota, indicated by a closer composition to the undisturbed community. The supplementation of individual prebiotics or a combination of 2’-FL and GOS could have added value in promoting the recovery of microbiota in the gut of antibiotic-treated infants. The recovery of microbial activity depended on the degree of perturbation, as this might rely on the presence or absence of metabolite producing bacteria and the cross-feeding activity. The supplementation of prebiotics might be beneficial in stimulating the cross-feeding of substrates and metabolites, although caution should be taken due to possible changes in the degrading capacity of disturbed microbiota.

## Data availability statement

The datasets presented in this study can be found in online repositories. The names of the repository/repositories and accession number(s) can be found at: EMBL-EBI accession number: PRJEB58807.

## Author contributions

ME, KV, and HS designed and planned the experiment. ME performed gut model experiment, laboratory analysis, and processing the sequencing data and drafted the manuscript. ME, KV, HS, DB, JP, and IA discussed the analysis plan. ME and DB performed statistical analysis and prepared the figures. HS and IA acquired funding. AN involved in discussion and provided prebiotics. CK involved in fecal materials collection and performed GOS analysis. KV, HAS, DB, JP, IA, AN, CK, and HS reviewed and contributed to the manuscript.

## Funding

This study was performed within the public/private partnership coordinated by the Carbohydrate Competence Center (CCC-Carbobiotics) - Grant number ALWCC.2017.011. CarboBiotics is jointly funded by the Dutch Research Council (NWO), FrieslandCampina, AVEBE, and NuScience.

## Conflict of interest

AN is employed by FrieslandCampina.

The remaining authors declare that the research was conducted in the absence of any commercial or financial relationships that could be construed as a potential conflict of interest.

## Publisher’s note

All claims expressed in this article are solely those of the authors and do not necessarily represent those of their affiliated organizations, or those of the publisher, the editors and the reviewers. Any product that may be evaluated in this article, or claim that may be made by its manufacturer, is not guaranteed or endorsed by the publisher.

## Supplementary material

Supplementary Figure S1 Microbiota type and composition of individual infant faecal slurries. **(A)** The dynamic change in microbiota type of faeces from each infant between 1 and 3 months after birth. **(B)** Microbiota composition of faeces used in pool faecal slurries. Faecal inoculum of 1 month group (1-M pool) was pooled from faecal slurries that showed community characterised with a mixed of taxa. While faecal inoculum of 3 months group (3-M pool) was pooled from faecal slurries that showed a community dominated by Bifidobacterium (> 50 %). Slurries A and B were collected from the same infant at different collection visits within the same week.

Supplementary Figure S2 Total bacterial 16S rRNA gene counts quantified by qPCR. Each treatment (with or without antibiotic or prebiotic) was run in duplicate or triplicate in TIM-2 colon model inoculated with pooled faeces from 1-month- (1-M colon model) and 3-months old (3-M colon model) infants. Average lines are shown for each treatment and the shaded areas represent standard error of the mean (SEM). Antibiotic pulses were indicated by red arrows. Significant differences were seen in the total bacterial 16S rRNA gene counts between the 1-M group treated with and without antibiotic at time point 52 h and 56 h according to Wilcoxon test.

Supplementary Figure S3 Microbiota composition (absolute abundance and proportion) of TIM-2 samples at ASV level. Samples were grouped by the simulated age of colon model (1- or 3-M) and treatment (with or without antibiotic, and with or without either prebiotic, GOS or 2’-FL). Each treatment was performed in duplicate or triplicate, with each run labelled A, B, or C. Antibiotics were added at 0, 8, 24 and 32 hours, immediately after sampling (indicated by red arrows).

Supplementary Figure S4 Number of observed genera **(A)** and effective Shannon diversity of genera **(B)** between microbiota treated with and without amoxicillin/clavulanate in TIM-2 samples at different time points. Antibiotics were added at 0, 8, 24 and 32 h, prior to sampling (indicated by red arrows). Wilcoxon test was used to compare the group with and without antibiotic. Significant differences are indicated by * (*p* < 0.05) and ** (*p* < 0.01).

Supplementary Figure S5 Ordination plots on log10-transformed absolute abundances of genera in microbiota from different treatments in TIM-2. The samples were grouped by the simulated age (1- or 3-M) of colon model and sampling time point. Antibiotic treated group showed dissimilarity to the untreated group from time point 24 h onwards in both age groups.

Supplementary Figure S6 PCoA of Generalized UniFrac distance (GUniFrac). The samples were grouped by the simulated age (1- or 3-M) of colon model and sampling time point. Antibiotic treated group showed dissimilarity to the untreated group from time point 24 h onwards but showing a closer microbiota at time point 72 h in both age groups.

Supplementary Figure S7 Heatmap showing the log10-transformed quantitative microbiota profiling (QMP) abundance of genera with absolute taxon weight of at least 0.5 on PRC response dimension 1, on 1-M microbiota. The abundances were grouped per time point for each treatment.

Supplementary Figure S8 Heatmap showing the log10-transformed quantitative microbiota profiling (QMP) abundance of genera with absolute taxon weight of at least 0.5 on PRC response dimension 1, on 3-M microbiota. The abundances were grouped per time point for each treatment.

Supplementary Figure S9 Production of microbial metabolites over time in each treatment run of 1-M and 3-M colon models. Cumulative production of acetate, propionate, butyrate, ammonia, lactate and propanol are shown. Luminal measurements of valerate, iso-butyrate, iso valerate, succinate, 1,2-propanediol, glucose, lactose and galactose are shown. Antibiotics were added at 0, 8, 24 and 32 h, prior to sampling (indicated by red arrows).

Supplementary Figure S10 Galacto-oligosaccharides (GOS) traces in antibiotic-treated TIM- 2 luminal and dialysis samples at time points 28 h and 32 h using LC-MS, following the method of Logtenberg et al. (2020). Vivinal GOS consists of mainly reducing isomers of DP 3 and DP 4. Some traces of GOS were detected in the luminal and dialysis samples of antibiotic-treated group at time points 28 h and 32 h, indicating incomplete degradation. GOS was either undetected or detected in small amount in the luminal samples of control treatment (without antibiotic) and GOS treatment (with antibiotic) at other time points (data not shown).

Supplementary Table S1 Linear regression model of taxa absolute abundances per time point.

Supplementary Table S2 Linear regression model of metabolite concentration per time point.
