# Supplementary figures and images for: Microbiota-dependent influence of prebiotics on the resilience of infant gut microbiota to amoxicillin/clavulanate perturbation in an in vitro colon model
Source: Front Microbiol. 2023 May 18;14:1131953. doi: 10.3389/fmicb.2023.1131953 (PMC10232780; doi:10.3389/fmicb.2023.1131953)

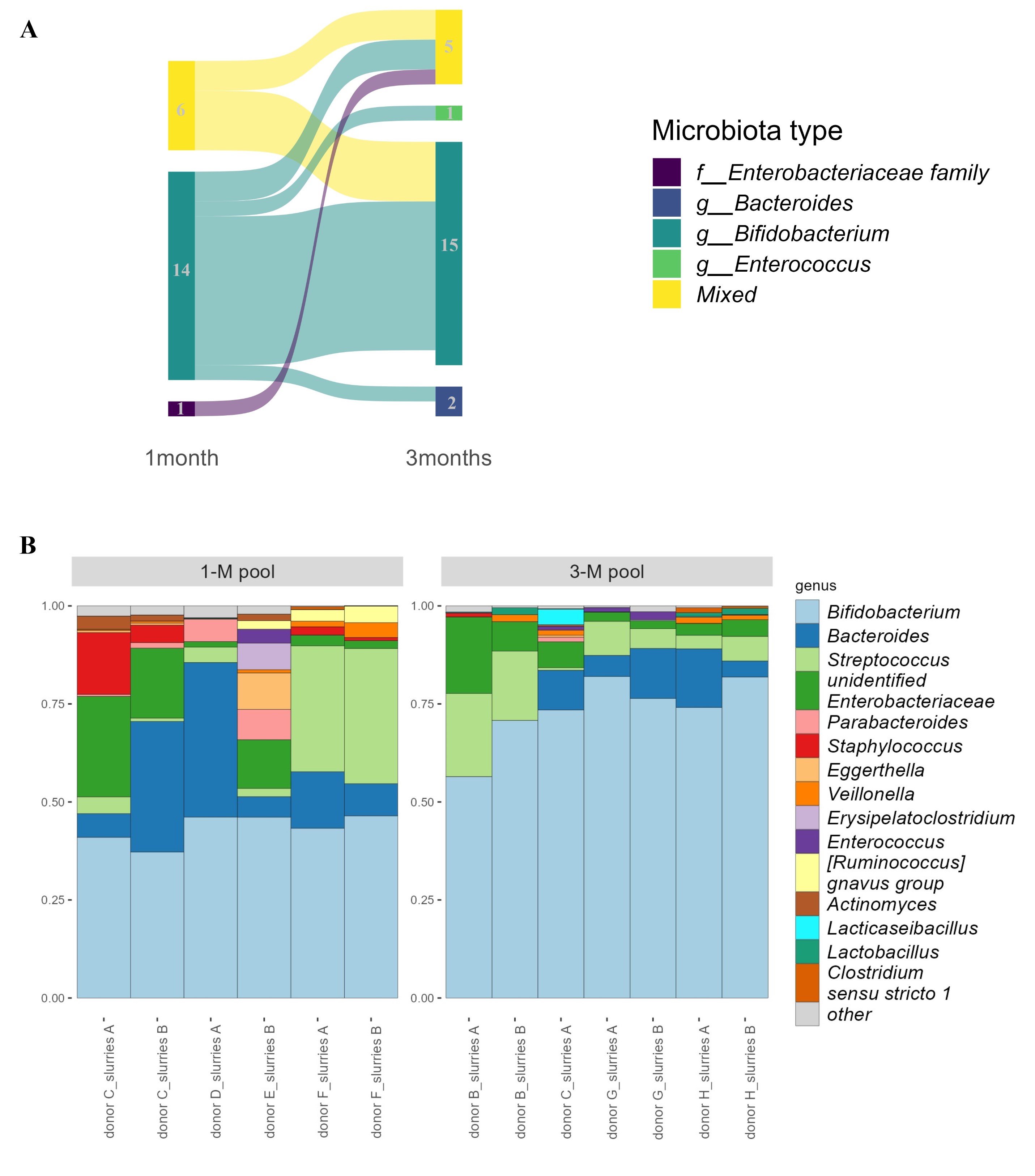

Supplement: Supplementary Figure S1 — Microbiota type and composition of individual infant faecal slurries. (A) The dynamic change in microbiota type of faeces from each infant between 1 and 3 months after birth. (B) Microbiota composition of faeces used in pool faecal slurries. Faecal inoculum of 1 month group (1-M pool) was pooled from faecal slurries that showed community characterised with a mixed of taxa. While faecal inoculum of 3 months group (3-M pool) was pooled from faecal slurries that showed a community dominated by Bifidobacterium (> 50 %). Slurries A and B were collected from the same infant at different collection visits within the same week. [file Image_1.JPEG]

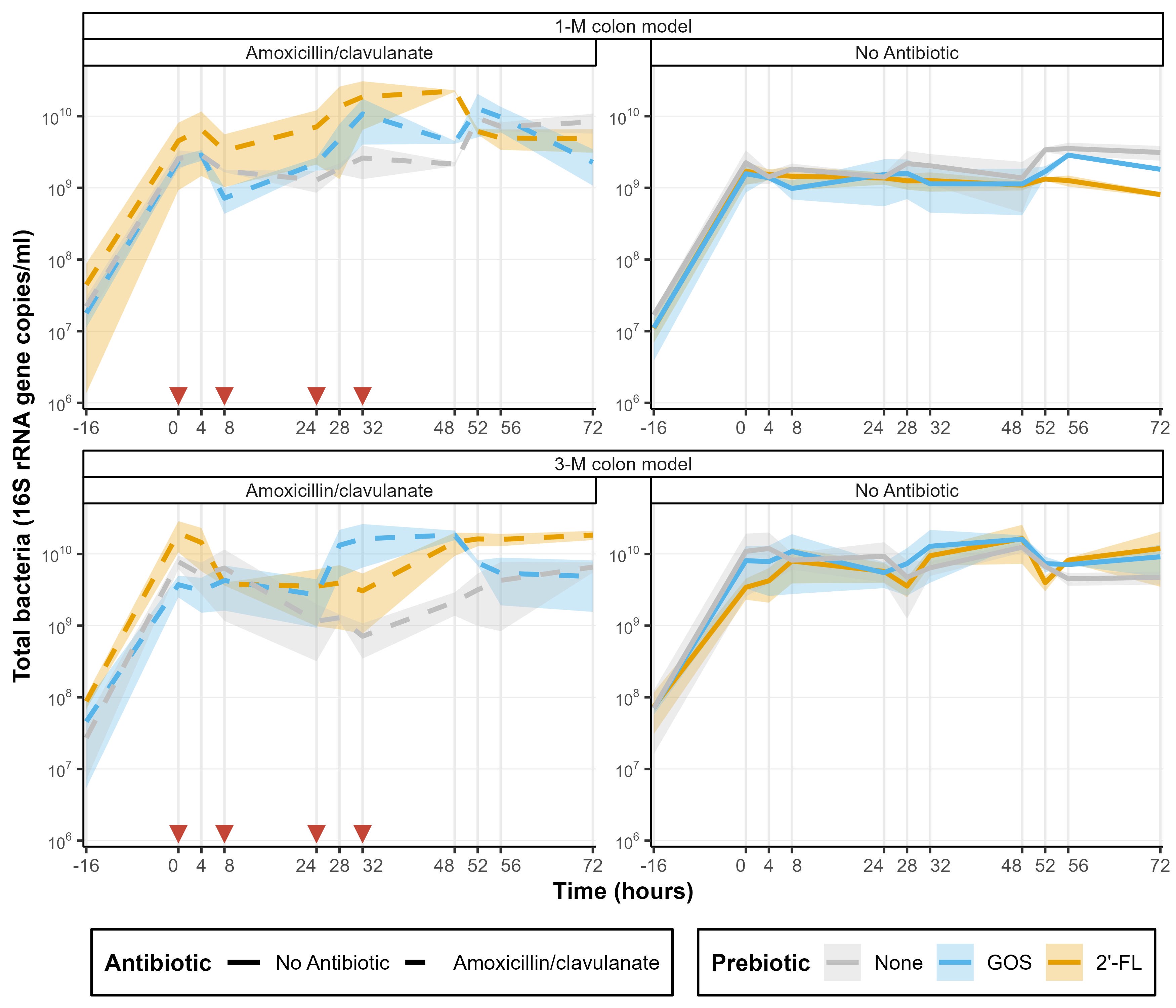

Supplement: Supplementary Figure S2 — Total bacterial 16S rRNA gene counts quantified by qPCR. Each treatment (with or without antibiotic or prebiotic) was run in duplicate or triplicate in TIM-2 colon model inoculated with pooled faeces from 1-month- (1-M colon model) and 3-months old (3-M colon model) infants. Average lines are shown for each treatment and the shaded areas represent standard error of the mean (SEM). Antibiotic pulses were indicated by red arrows. Significant differences were seen in the total bacterial 16S rRNA gene counts between the 1-M group treated with and without antibiotic at time point 52 h and 56 h according to Wilcoxon test. [file Image_2.JPEG]

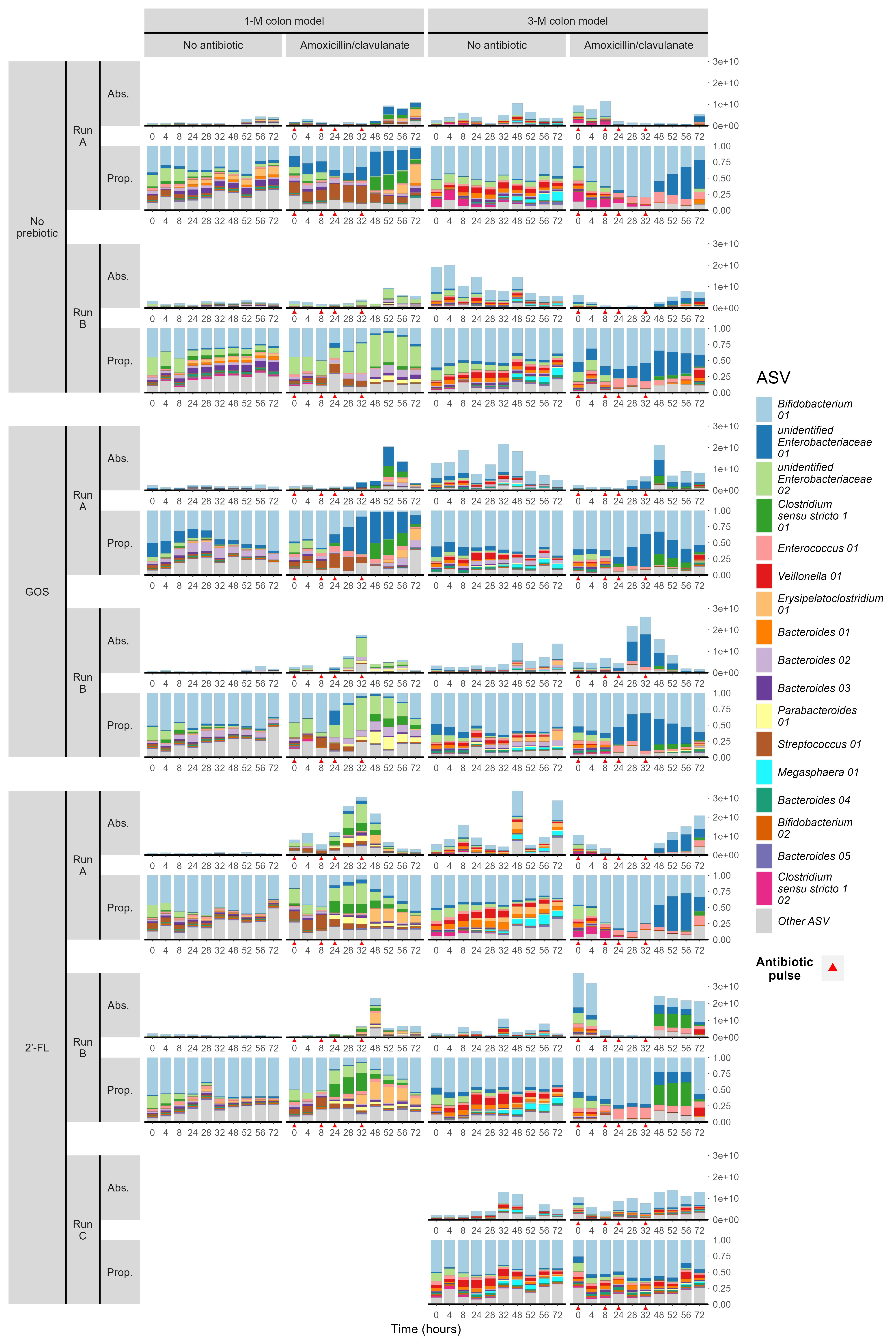

Supplement: Supplementary Figure S3 — Microbiota composition (absolute abundance and proportion) of TIM-2 samples at ASV level. Samples were grouped by the simulated age of colon model (1- or 3-M) and treatment (with or without antibiotic, and with or without either prebiotic, GOS or 2’-FL). Each treatment was performed in duplicate or triplicate, with each run labelled A, B, or C. Antibiotics were added at 0, 8, 24 and 32 hours, immediately after sampling (indicated by red arrows). [file Image_3.JPEG]

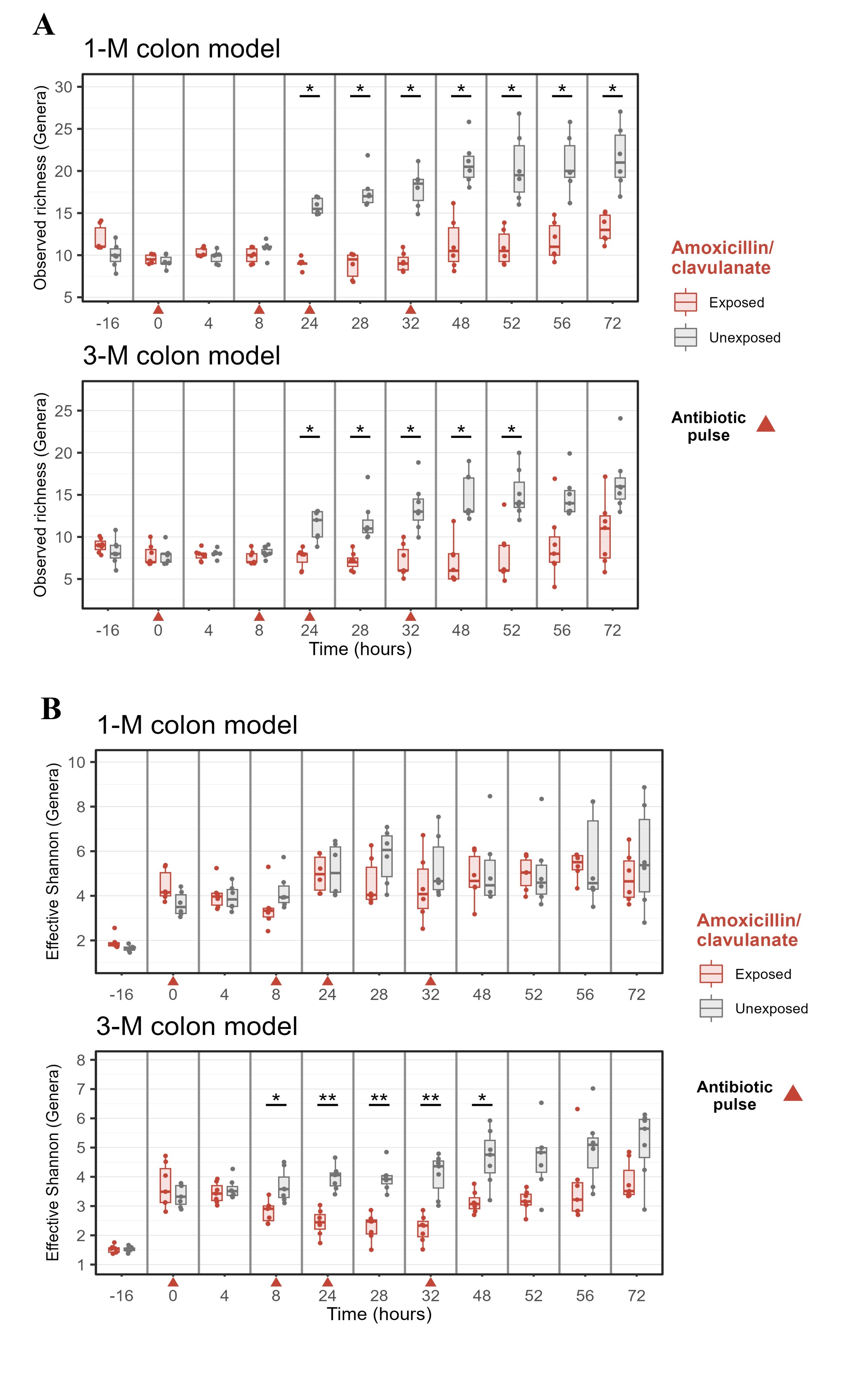

Supplement: Supplementary Figure S4 — Number of observed genera (A) and effective Shannon diversity of genera (B) between microbiota treated with and without amoxicillin/clavulanate in TIM-2 samples at different time points. Antibiotics were added at 0, 8, 24 and 32 h, prior to sampling (indicated by red arrows). Wilcoxon test was used to compare the group with and without antibiotic. Significant differences are indicated by * (p < 0.05) and ** (p < 0.01). [file Image_4.JPEG]

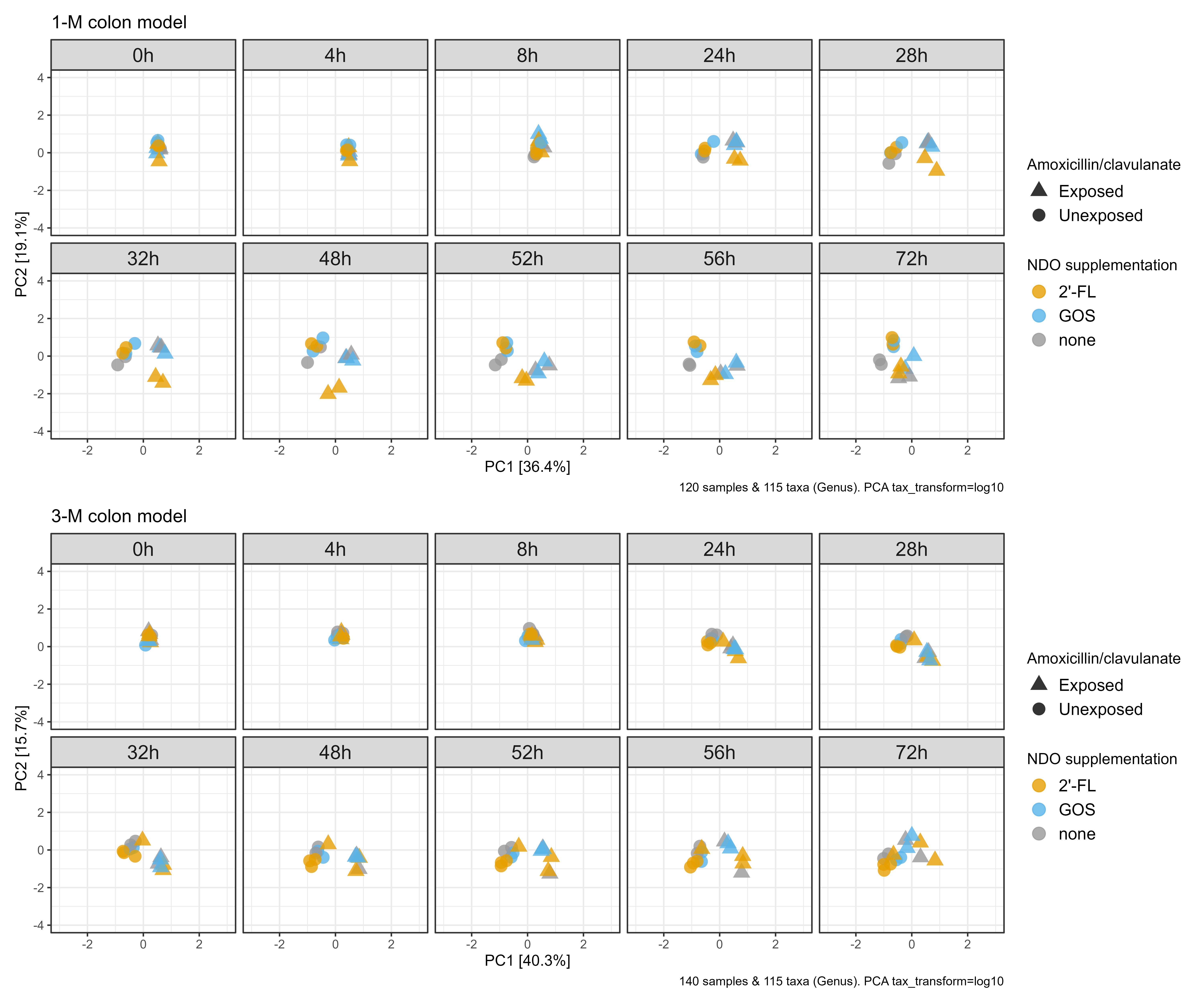

Supplement: Supplementary Figure S5 — Ordination plots on log10-transformed absolute abundances of genera in microbiota from different treatments in TIM-2. The samples were grouped by the simulated age (1- or 3-M) of colon model and sampling time point. Antibiotic treated group showed dissimilarity to the untreated group from time point 24 h onwards in both age groups. [file Image_5.JPEG]

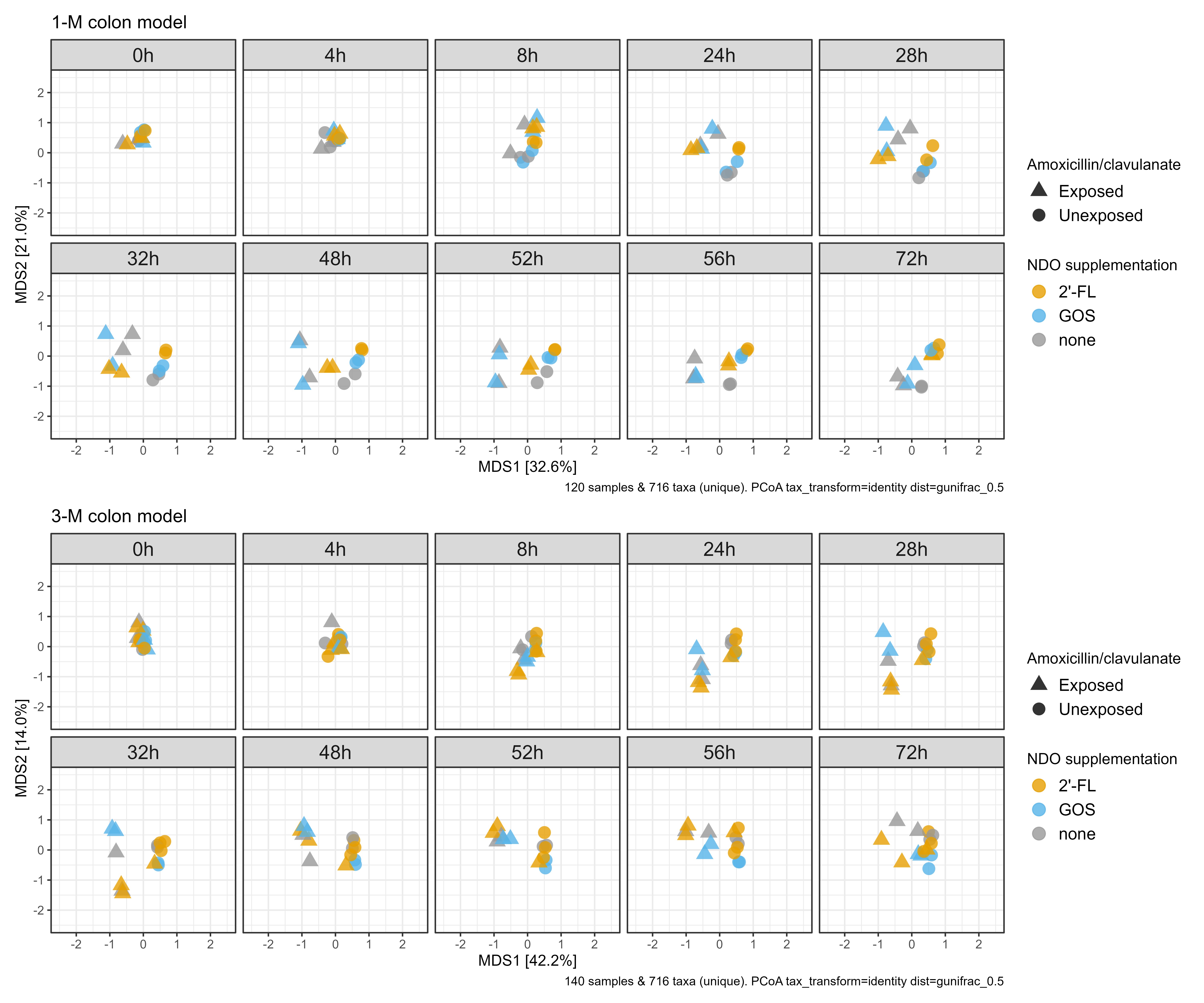

Supplement: Supplementary Figure S6 — PCoA of Generalized UniFrac distance (GUniFrac). The samples were grouped by the simulated age (1- or 3-M) of colon model and sampling time point. Antibiotic treated group showed dissimilarity to the untreated group from time point 24 h onwards but showing a closer microbiota at time point 72 h in both age groups. [file Image_6.JPEG]

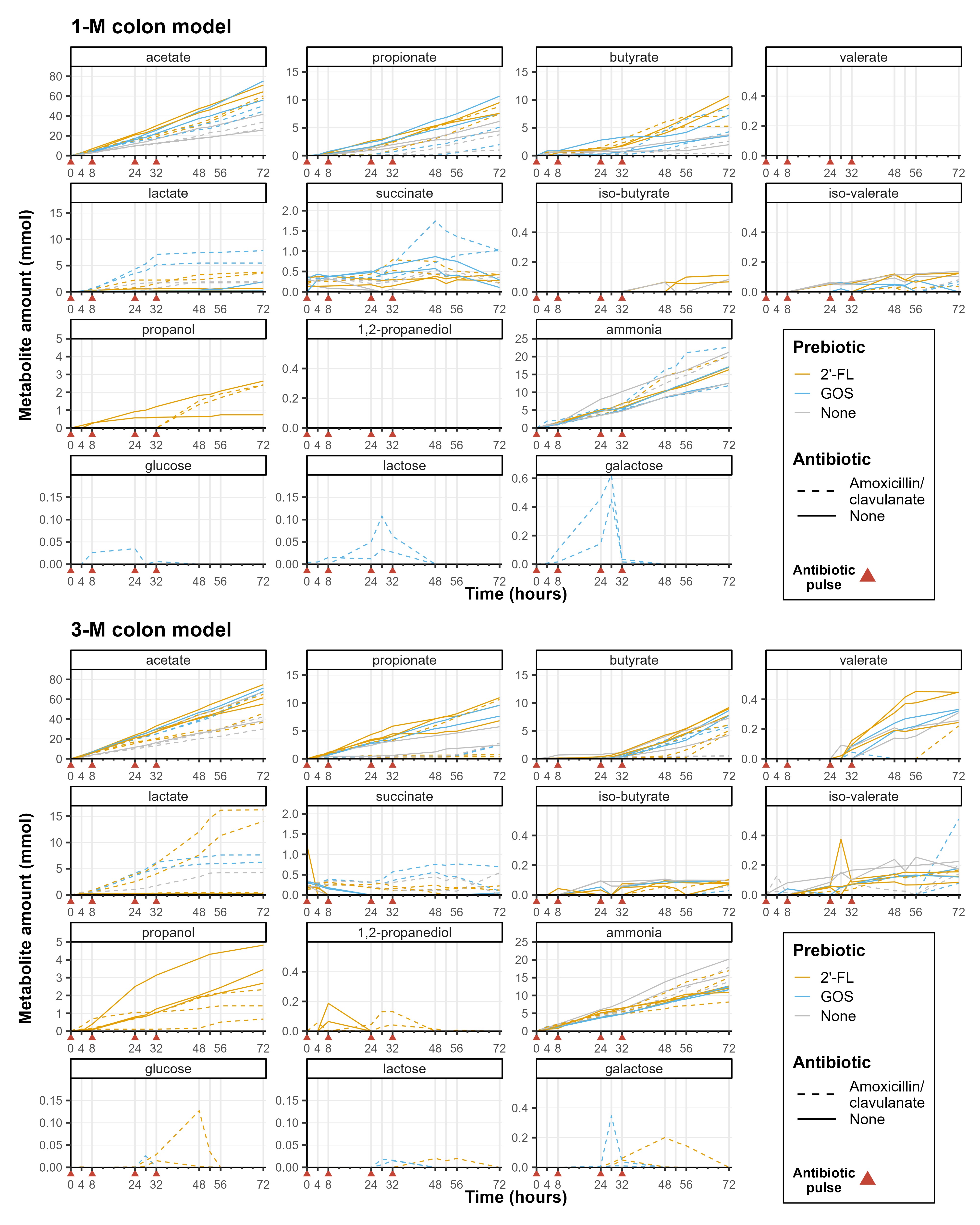

Supplement: Supplementary Figure S9 — Production of microbial metabolites over time in each treatment run of 1-M and 3-M colon models. Cumulative production of acetate, propionate, butyrate, ammonia, lactate and propanol are shown. Luminal measurements of valerate, iso-butyrate, iso valerate, succinate, 1,2-propanediol, glucose, lactose and galactose are shown. Antibiotics were added at 0, 8, 24 and 32 h, prior to sampling (indicated by red arrows). [file Image_9.JPEG]

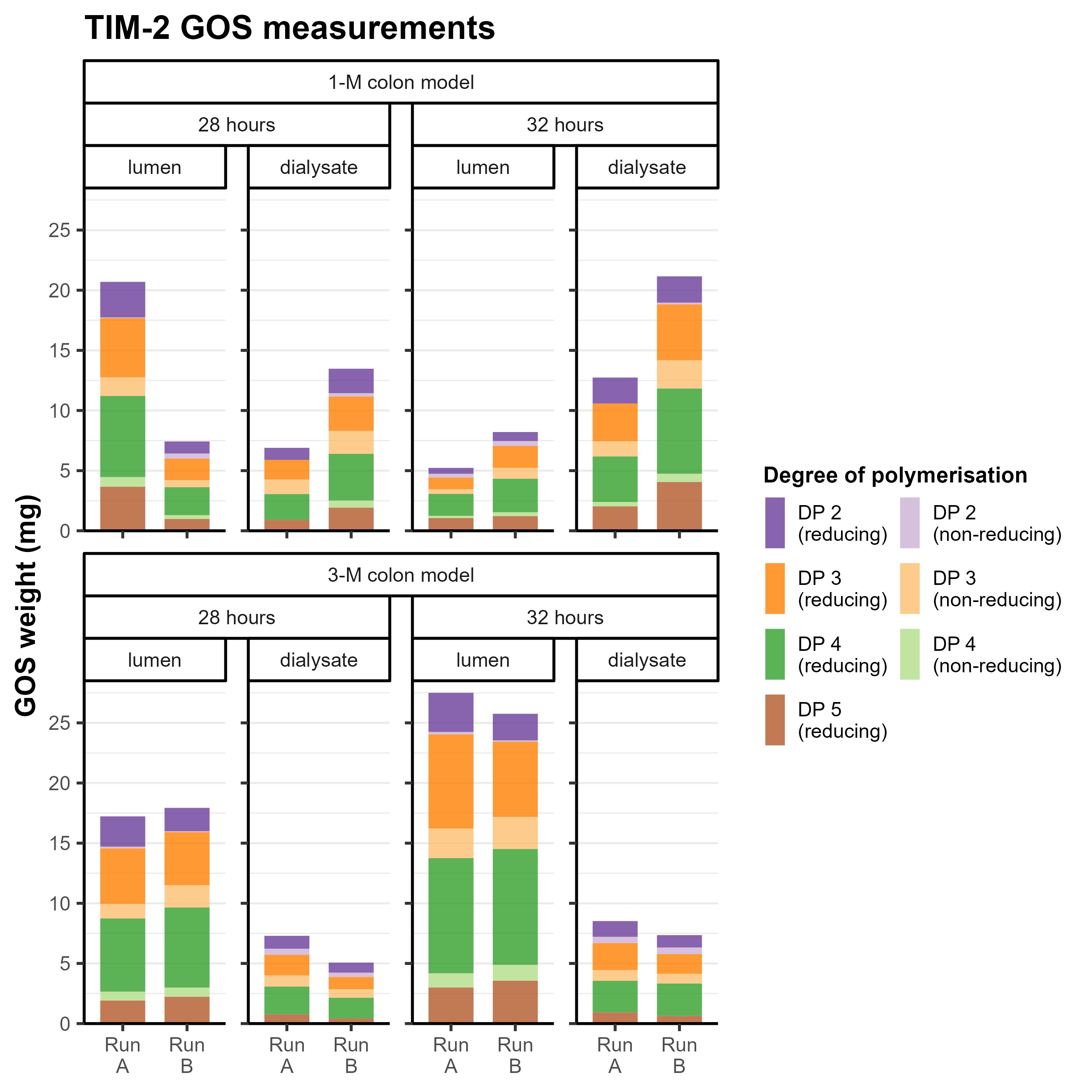

Supplement: Supplementary Figure S10 — Galacto-oligosaccharides (GOS) traces in antibiotic-treated TIM- 2 luminal and dialysis samples at time points 28 h and 32 h using LC-MS, following the method of Logtenberg et al. (2020). Vivinal GOS consists of mainly reducing isomers of DP 3 and DP 4. Some traces of GOS were detected in the luminal and dialysis samples of antibiotic-treated group at time points 28 h and 32 h, indicating incomplete degradation. GOS was either undetected or detected in small amount in the luminal samples of control treatment (without antibiotic) and GOS treatment (with antibiotic) at other time points (data not shown). [file Image_10.JPEG]
